# Salivary microbiome of healthy women of reproductive age

**DOI:** 10.1128/mbio.00300-23

**Published:** 2023-09-01

**Authors:** Eline Cauwenberghs, Eline Oerlemans, Stijn Wittouck, Camille Nina Allonsius, Thies Gehrmann, Sarah Ahannach, Ilke De Boeck, Irina Spacova, Peter A. Bron, Gilbert Donders, Veronique Verhoeven, Sarah Lebeer

**Affiliations:** 1 Department of Bioscience Engineering, Research Group Environmental Ecology and Applied Microbiology, University of Antwerp, Antwerp, Belgium; 2 Department of Obstetrics and Gynaecology, University Hospital Antwerp, Edegem, Belgium; 3 Regional Hospital Heilig Hart, Tienen, Belgium; 4 Femicare, Clinical Research for Women, Tienen, Belgium; 5 Department of Family medicine and population health (FAMPOP), University of Antwerp, Antwerp, Belgium; Columbia University, New York, New York, USA

**Keywords:** human microbiome, salivary microbiome, women, conserved, hormonal contraception

## Abstract

The human salivary microbial community plays a crucial role in local and systemic diseases. Biological and lifestyle factors such as menstrual cycle, oral hygiene, and smoking have been documented to impact this community. However, while hormonal contraceptives are the most prescribed drug in healthy women and intimate partners play key roles in microbial exchange between humans, their impact on the salivary microbiome of women of reproductive age have been understudied. Additionally, the role of other lifestyle factors such as diet, allergies, age, and stress on the saliva microbiome of the general population is not well understood. Here, we studied the salivary microbiome of 255 healthy women of reproductive age using self-sampling kits and 16S rRNA amplicon sequencing combined with questionnaires on lifestyle and host-related parameters. A preserved salivary bacterial community of 12 genera (*Actinobacillus, Actinomyces, Alloprevotella, Campylobacter, Fusobacterium, Gemella, Granulicatella, Leptotrichia, Neisseria, Prevotella, Streptococcus,* and *Veillonella*) was identified. Contrary to what we expected, the number of intimate partners or specific contraceptive use did not have a major impact on these bacterial communities. However, recent use of oral antibiotics was associated with a significant decrease in richness at genus level and increase in mean relative abundances of several taxa. Being stressed or nervous was associated with a significantly increased richness of the salivary microbiome at the level of amplicon sequencing variants . Nevertheless, these associations with host-related and lifestyle variables only appeared to be subtle, suggesting that the salivary microbiome is mainly driven by the buccal environment and health status of an individual.

**IMPORTANCE:**

The salivary microbiome has been proven to play a crucial role in local and systemic diseases. Moreover, the effects of biological and lifestyle factors such as oral hygiene and smoking on this microbial community have already been explored. However, what was not yet well understood was the natural variation of the saliva microbiome in healthy women and how this is associated with specific use of hormonal contraception and with the number of different sexual partners with whom microbiome exchange is expected regularly. In this paper, we characterized the salivary microbiome of 255 healthy women of reproductive age using an in-depth questionnaire and self-sampling kits. Using the large metadata set, we were able to investigate the associations of several host-related and lifestyle variables with the salivary microbiome profiles. Our study shows a high preservation between individuals.

## INTRODUCTION

The oral cavity forms a complex microbial habitat due to its various anatomical sites, including the tongue, cheek, throat, and saliva (1). Saliva exhibits a stable temperature of 37°C and stable pH between 6.5 and 7.5, which is preferred by most bacterial species (2). It is an important bacterial reservoir for other niches such as the gut (3) and airways (4). Microorganisms can be introduced into the mouth through, for example, breathing, drinking, eating, and kissing. The latter was investigated in a controlled experiment where salivary microbiota of 21 couples were evaluated before and after intimate kissing (5). The authors found clear correlations between the similarity indices of the salivary microbiota of couples and self-reported kissing frequencies. Thus, intimate partners play a key role in microbial exchange, but it is as present not well understood whether intimate partners are of importance in shaping the human microbiome of healthy adults. Moreover, it is not known how long the effect of kissing lasts on salivary microbial communities and whether people with multiple partners have a different diversity or ecological architecture of the salivary microbiome.

Relevant associations between differences in diversity measures or (relative) abundance of individual microbial taxa in the salivary microbiome and various host factors, including local and systemic human diseases such as caries (6, 7), obesity (8, 9), and cardiovascular disease (10), have been mapped in different studies. Additionally, biological and lifestyle factors such as good oral hygiene (11, 12), smoking (13 – 15), and medication use such as thyroid hormones and statins (16) or oral antibiotics (17, 18) have been shown to be of importance for the constellation of the oral microbiome. To women specifically, the influence of hormonal contraception on the salivary microbiome is of great interest as this is one of the most prescribed medication for women of reproductive age (19). However, the impact of hormonal contraception on the microbiome at other human body sites than the vagina is currently understudied. In addition, it is not yet well understood how environmental and lifestyle factors such as diet, allergies, and stress impact the (saliva) microbiome in the general population.

Here, we characterized the salivary microbiome of overall healthy women of reproductive age (*n* = 255, 18–53 years old) in Western Europe who were recruited outside a clinical environment via a citizen-science approach as part of the Isala study in Belgium (https://isala.be/en/). Women were recruited based on intimate partnership, contraception use, and age. Using self-sampling kits and an extensive questionnaire related to type of contraceptive use, sexual lifestyle, physical and mental health, food intake, lifestyle factors, and hygiene habits, resulted in a large compendium of microbiome data and metadata. Women donated samples from their vaginal, skin, and saliva microbiome. The results of the saliva microbiome are reported here.

## MATERIALS AND METHODS

### Citizen science cohort

The protocol of this study was in accordance with the Declaration of Helsinki. The study was approved by the ethical committee of the Antwerp University Hospital/University of Antwerp (Belgium, B300201942076) and registered on clinicaltrials.gov (NCT04319536). In March 2020, the Isala project, a citizen-science project on women’s health was launched in Belgium (https://isala.be/en) (20). A media call for female participants resulted in a cohort of 3,345 women who were willing to donate samples from different human body sites for microbiome research and provided metadata on multiple biological, lifestyle, and environmental factors. From this large cohort of Isala participants, 293 healthy women were selected to donate a saliva sample. This selection encompasses balanced groups in terms of number of sexual partners, frequency of vaginal intercourse, contraception use, and age, which were the primary factors of interest for this study. In addition, other aspects of general, sexual, and mental health were collected via questionnaires (Supplementary file A). After written informed consent, the participants received a kit containing all necessary materials and instructions for self-sampling, among others an eNAT (Copan, Brescia, Italy) swab and a sterile container (Carl Roth, Karlsruhe, Germany).

### Data collection and bacterial DNA extraction from saliva samples

To minimize variation due to hormonal fluctuation, participants were asked to provide an unstimulated saliva sample at the luteal phase in their menstrual cycle (approximately 19 days after the start of monthly bleeding). If participants did not have a traceable cycle [e.g., due to a hormonal intrauterine device (IUD)], they were unrestricted to when to start their sampling. First thing in the morning, participants collected saliva in a sterile container after which they turned the swab around in the saliva to acquire sufficient biomass, followed by deposition in a sterile container containing eNAT transport buffer and storage at home in the fridge (approximately 4°C). Notable, the eNAT Specimen Collection and Transport system contains cotton swabs that are typically used for swabbing of mucosal surfaces. However, participants were specifically instructed to only bring the swab in the collected unstimulated saliva to avoid mucosal contamination. This method was already validated for 16S rRNA sequencing (21) and has shown to produce consistent microbiome profiles compared to other sampling procedures (22). Subsequently, samples were transported at room temperature to the University of Antwerp via the national parcel service (Bpost) and immediately stored at −20°C upon arrival prior to further processing (20). Prior to DNA extraction, all samples were vortexed for 15–30 seconds. Five hundred microliters of the eNAT buffer was used for manual extraction using the DNeasy PowerSoil Pro Kit (Qiagen, Hilden, Germany). DNA extraction was performed according to the manufacturer’s instructions. Negative extraction and PCR controls were included at regular time points throughout the study. DNA concentrations were measured using the Qubit 3.0 Fluorometer according to the manufacturer’s instructions (Life Technologies, Ledeberg, Belgium).

### Illumina MiSeq 16S rRNA gene amplicon sequencing

To determine the taxonomic composition of the bacterial communities, Illumina MiSeq 16S rRNA gene amplicon sequencing was performed on amplicons generated using extracted DNA from the saliva swabs as templates. An in-house optimized protocol was followed, as described in Lebeer et al. (20). Briefly, dual-index paired-end sequencing of the V4 region of the 16S rRNA gene was performed on a MiSeq Desktop Sequencer (M00984, Illumina).

Processing and quality control of the reads were performed using the R package DADA2 version 1.6.0 (23). Taxa and sample quality control were performed as described in Lebeer et al. (20). Two hundred fifty-five saliva samples were sequenced, of which 246 passed quality control based on estimated DNA concentrations. Further processing of the amplicon sequencing variant (ASV) table, ASV annotation (e.g., classification), sample annotation (metadata), data visualization, and statistical analysis were performed in R version 4.0.3 (R Core Team, 2018) using the in-house package tidyamplicons version 0.2.1 (publicly available at github.com/SWittouck/tidyamplicons) as described in De Boeck et al. (24). Alpha- and beta-diversity analyses were also performed as described before in De Boeck et al. (24).

### Large-scale questionnaire

Together with taking the saliva samples, all participants were asked filled out an online questionnaire on the Qualtrics platform (Qualtrics, Provo, UT, USA). Questions regarding general health, hygiene, and food intake were included. Body mass index (BMI) was calculated, and participants were divided into underweight group (BMI <18.5), normal weight group (BMI = 18.5–24.9), overweight group (BMI = 25–29.9), and obesity group (BMI ≥30). This was done based on the CDC Growth Charts as described in Callahan et al. (25). Questions regarding mental health were asked using a likert scale (scoring from 1 to 6). Less/more groups were created for all feelings by categorizing a scoring of 1–3 as “less” and 4–6 as “more.” The questionnaire can be found in the Supplementary Information 1.

### Statistical analysis

Quality control and processing of 16S rRNA amplicon reads were performed as described in Ahannach et al. (21). A total of 4,596,282 high-quality reads were generated. The reads were not trimmed but reads with more than two expected errors were removed. Next, forward and reverse reads were denoised per sample after which reads were merged after which read pairs with sequence conflicts were removed. Afterwards, chimeras were detected and removed with the removeBimeraDenovo function. Merged and denoised reads were taxonomically annotated from the phylum to the genus level with the assignTaxonomy function using EZBioCloud reference 16S rRNA database (26). Non-bacterial ASVs (e.g., mitochondria and chloroplasts) and ASVs with a length greater than 299 bases were removed. On average, ASVs had an average length of 253 bp. After filtering, there were 4,595,697 total reads and the samples contained on average 20,157 high-quality reads per sample. For analyses at the genus level, ASV read counts were aggregated at the genus level or at the most specific level at which taxonomic annotation was available. If an exact match was found with exactly one species in the reference database, this is indicated throughout the manuscript as “most likely to be.” Processing of the ASV table, classification and sample annotation (metadata) as well as data analysis was performed in R v4.0.1 (27) using the package tidyverse version 1.3.0 (28) and the in-house package tidyamplicons version 0.2.2 (github.com/SWittouck/tidyamplicons).

To investigate how covariates confounded one another, two analyses were performed. Firstly, covariates were correlated using Pearson correlation and secondly, the association of the covariates with the first six principle components of the Bray-Curtis beta-diversity matrix of the abundance data were analyzed using simple linear regression to identify confounding variables to add to association analyses. Associations of individual bacterial taxa with participant covariates were assessed to give an overview of which covariates are linked with changes in relative abundances of certain genera/ASVs and was done as followed. To ensure that batch effects and interpersonal variation were accounted for in our models, we identified age, BMI, and read concentration as possible confounders. ASV counts were aggregated at the genus and family levels. At each level, only taxa which were present (i.e., non-zero counts) in at least 10% of all samples were retained. For each factor of interest, we defined a model *taxa ~factor + BMI +age + readconcentration +readcount*. Each model was tested using six different differential abundance tests: aldex2 (29), ancombc (30), DESeq2 (31), limma (32), maaslin2 (33), and a linear regression on the centered-log-ratio transformed relative abundances of individual taxa abundances. For each taxa-factor association test, we report the number of tools that reported a significant association (consensus). We consider a taxa-factor association significant only if at least three tools find a significant effect following Benjamini-Hochberg correction for the False Discovery Rate (*q* < 0.05). This functionality is implemented in the multidiffabundance R package (https://github.com/thiesgehrmann/multidiffabundance).The analyses can be found in Table S2 to S4 for ASV, genus, and family levels, respectively. Furthermore, we tested the effects on beta-diversity using a permutational multivariate analysis of variance (PERMANOVA) test [adonis2 function in vegan R package (34)] and on (Shannon) alpha-diversity using a linear regression, in both instances with age, BMI, and readconcentration as covariates. However, at these levels, there were no significant effects.

## RESULTS

### Identification of a core salivary bacterial microbiome in the Isala cohort

**Fig 1:**
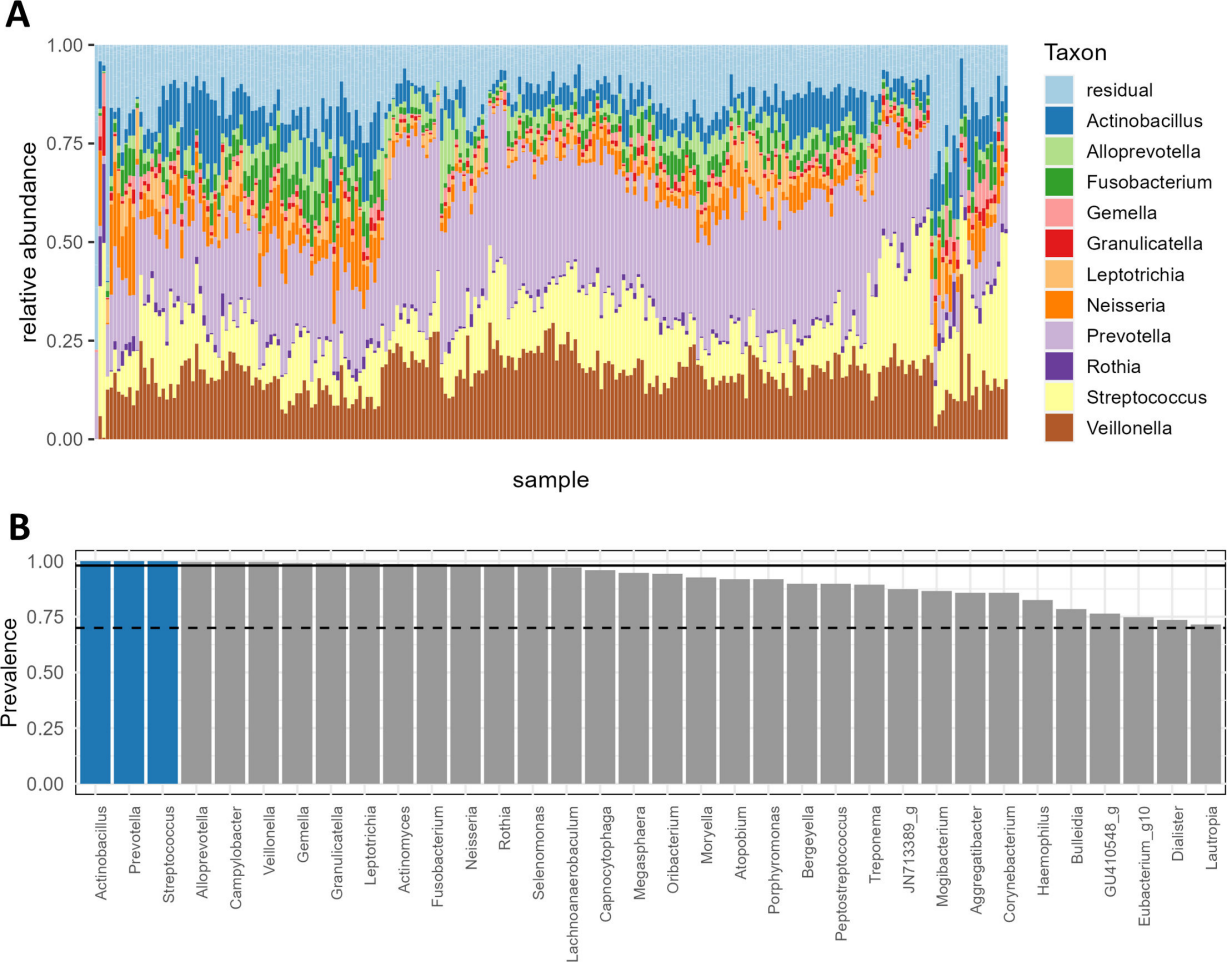
Overview of the salivary microbiome composition and preservation of genera in our female cohort. (**A**) Stacked bar chart describing the microbial composition of all participants in terms of the relative abundance of the 11 most abundant genera. Samples are ordered according to an average-linkage hierarchical clustering based on Bray-Curtis distances. (**B**) Bar plot showing the preservation of genera in our cohort: all genera with a prevalence of at least 70% are shown. The full and dotted lines indicate a prevalence of 98% and 70%, respectively. Blue represents omnipresent genera.

Two hundred and ninety-three women were selected from the large cross-sectional Isala cohort (20). Women were selected based on age, contraception use, number of intimate partners, and frequency of sexual intercourse to maximize statistical power for the detection of possible associations between these key lifestyle and prescription drug factors of interest (https://isala.be). The other lifestyle and environmental factors were not controlled but monitored via questionnaires. Of the 293 selected women, 246 participants provided a saliva sample and 228 participants (78.08%) provided both their saliva sample and metadata, and all samples passed the quality control. There was no bias based on age, BMI, and smoking between participants who provided metadata (responders) and those who did not (non-responders) (Table S1). This cohort was characterized by a mean age of 31.5 ± 7.8 years with 98 women (42.98%) in the 18–28 age group, 82 (35.96%) in the 29–39 age group, and 47 (20.61%) in the 40–50 age group. In terms of contraceptive method, 32.02% used a combination pill, 32.02% had a hormonal IUD, and 34.65% of the participants did not use any hormonal contraception (including copper IUD). The remainder of participants (1.32%) changed contraception method after inclusion and used another type of hormonal contraception such as a progesterone-only pill or vaginal ring. Precisely 66.67% of the participants had one sexual partner, 21.93% indicated to have more than one partner, and 11.40% did not have a partner. Participants’ demographics (*n* = 228) and general information regarding (oral) health, contraception and diet is presented in Table 1. All participants self-reported to be generally healthy. The communities of the 246 female saliva samples showed to be mainly represented by the genera *Prevotella, Veillonella, Streptococcus, Actinobacillus,* and *Neisseria*, with mean relative abundances of 27.3%, 16.6%, 14.6%, 7.3%, and 4.8%, respectively (Fig. 1A). Hierarchical clustering of the microbial profiles at genus level indicated no discrete clusters. Twelve genera had a prevalence of at least 98%, namely *Actinobacillus, Actinomyces, Alloprevotella, Campylobacter, Fusobacterium, Gemella, Granulicatella, Leptotrichia, Neisseria, Prevotella, Streptococcus,* and *Veillonella* (Fig. 1B). *Actinobacillus*, *Prevotella,* and *Streptococcus* were omnipresent.

**TABLE 1:** Participants’ demographics and general information^*b*^

	Total (*n* = 228)	
	**[*n*]**	**[%]**
General		
Age (years)		
Range	19–53	/
Mean ± SD	31.5 ± 7.8	/
Body Mass Index (BMI, kg/m²) [mean ± SD]	24.1 ± 4.6	/
Obesity (BMI ≥30)	27	11.84
Underweight (BMI <18.5)	5	2.19
Born in Belgium	216	94.74
Current smoker	16	7.02
Oral antibiotic in last 3 mo	9	3.95
Kissed last 24 h	35	15.35
HPV vaccinated	92	40.35
Oral health		
Bleeding gums	25	10.96
Went to the dentist in the last week	13	5.70
Went to the dentist in the last year	192	84.21
Blood type		
O+	91	39.91
O–	24	10.53
A+	67	29.39
A–	11	4.82
B+	17	7.46
B–	1	0.44
AB+	5	2.19
Current sexual partners		
No partner	26	11.40
One partner	152	66.67
Multiple partners	50	21.93
Contraception		
Combination pill	73	32.02
Hormonal intrauterine device (IUD)	73	32.02
No hormonal contraception	79	34.65
Other	3	1.32
Diet		
Vegetarian^*a*^	170	88.08
Pescatarian^*a*^	11	5.70
Omnivore^*a*^	12	6.21
Vegan	15	6.58
Lactose intolerant	15	6.58
Gluten allergy	2	0.88
Respiratory health		
Respiratory allergies	34	14.91
Respiratory infection in the last week	11	4.82
Mental health		
Happy	181	79.39
Calm	158	69.30
Sad	31	13.60
Nervous	53	23.25
Stressed	81	35.53
Nothing to cheer up	181	79.39

^*a*^ These results are based on all participants who filled in the Food Frequency Questionnaire (FFQ, *n* = 193).

^*b*^ This information is based on all participants that filled in the questionnaire, but not necessarily sent in a saliva sample.

### The salivary microbiome is resilient toward variations in hormonal contraceptives and intimate partnership

**Fig 2:**
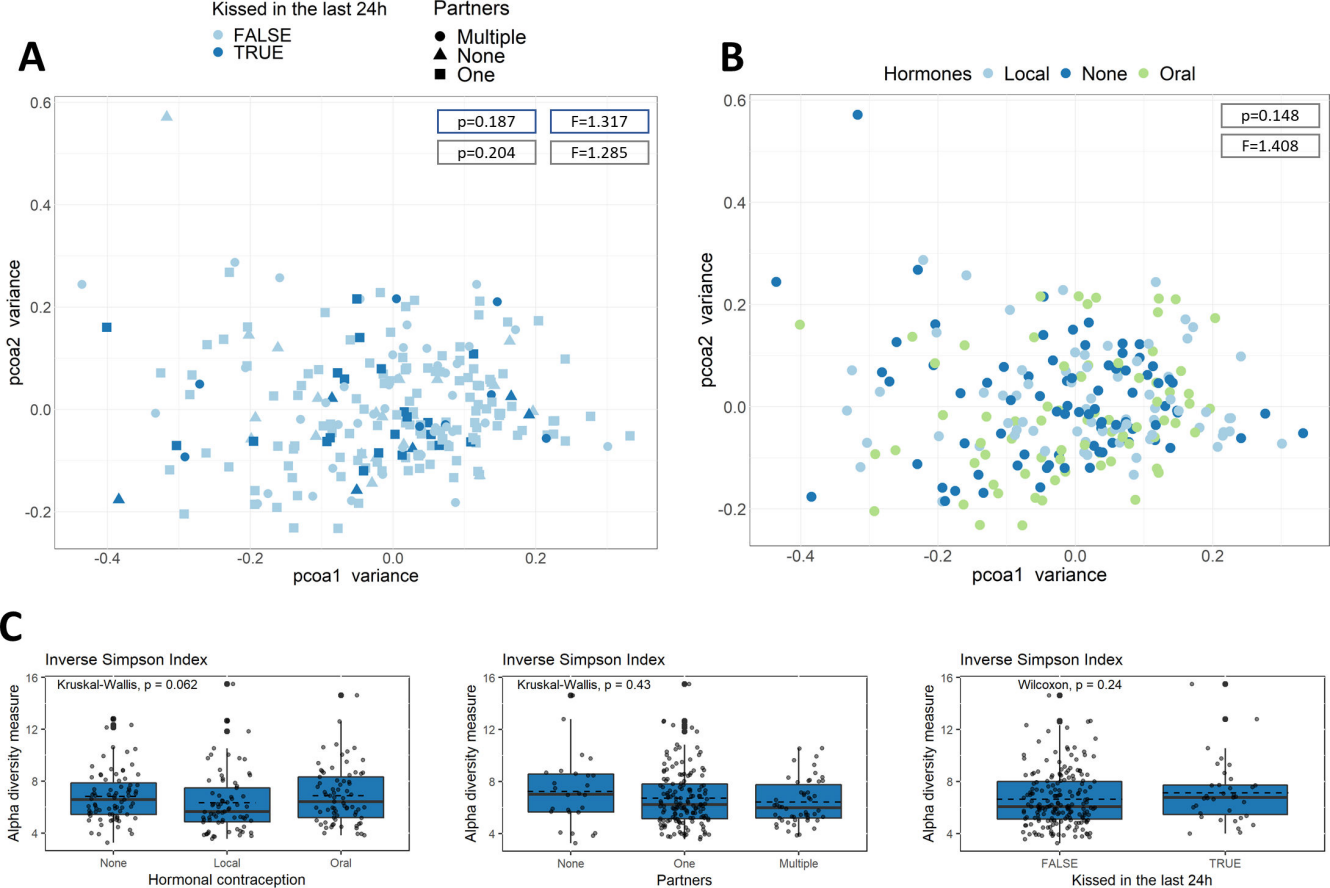
Statistical analysis of the association of hormones and sexual behavior with the beta- and alpha-diversity of the salivary microbiome. (**A**) PCoA plot distributing the samples according to beta-diversity (Bray-Curtis distance). Samples are colored by the use of hormonal contraception and shapes indicate the number of partners (none, one, or multiple). *P*-values and *F*-values (based on PERMANOVA) are indicated. Alpha-diversity determined by Inverse Simpson index for hormonal contraception (**B**) and number of partners (**C**). Dotted line represents the mean alpha-diversity.

We then looked for possible associations between the salivary microbiome and host factors. First, we investigated whether the variables were (negatively) correlated to one another (Fig. S1). Only variables that were defined in terms of each other (e.g., use of combination pill vs use of IUD) were correlated. The other variables were not highly correlated, so that we did not correct for this in downstream analysis. We then investigated the association of our predefined lifestyle factors of interest, i.e., intimate behavior [number of intimate partners (none, one or multiple)] and kissing in the last 24 hours, as well as the contraceptive method [no mechanical hormones, combined hormonal contraception (oral) and IUD contraception (local)] with the beta- and the alpha-diversity of the salivary microbiome. These variables did not show a significant association with the beta-diversity [as calculated by permutational multivariate analysis of variance (PERMANOVA)], with *P*-values of 0.187 (F = 1.317), 0.204 (F = 1.285), and 0.148 (F = 1.408) for kissing in the last 24 hours, number of partners, and contraception method, respectively (Fig. 2A and B). We also did not observe a significant associations for alpha-diversity, based both on the richness (Fig. S2) as well as Inverse Simpson (Fig. 2C). More specifically, for sexual behavior (partners and kissing), we calculated *P*-values of 0.43 and 0.92 for number of partners and 0.54 and 0.24 for kissing in the last 24 hours based on richness and Inverse Simpson, respectively. However, we did observe a near-significant decrease in alpha-diversity in participants who used an intrauterine hormonal contraceptive (IUD), based on Inverse Simpson Index (*P* = 0.06). When we investigated the association between hormonal contraception and the relative abundance of specific genera/ASVs in the salivary microbial composition (genus level in Fig. 4, ASV level in Fig. S4), we observed a decrease in the genera *Mitsuokella* and *Kingella* and *Capnocytophaga* ASV2 and *Prevotella* ASV10 for participants who use hormonal contraception when correcting for multiple comparisons based on one tool. Taken all these data together, in contrast to what we hypothesized mainly based on possible impact on the vaginal microbiome, in our selected cohort, sexual behavior and contraception method did not have a major impact on the salivary microbiome. All results of these analyses, including *P*-values corrected for multiple testing for each tool are shown in Tables S2 and S3 on ASV and genus level, respectively.

### The impact of oral health and antibiotics on the salivary microbiome

**Fig 3:**
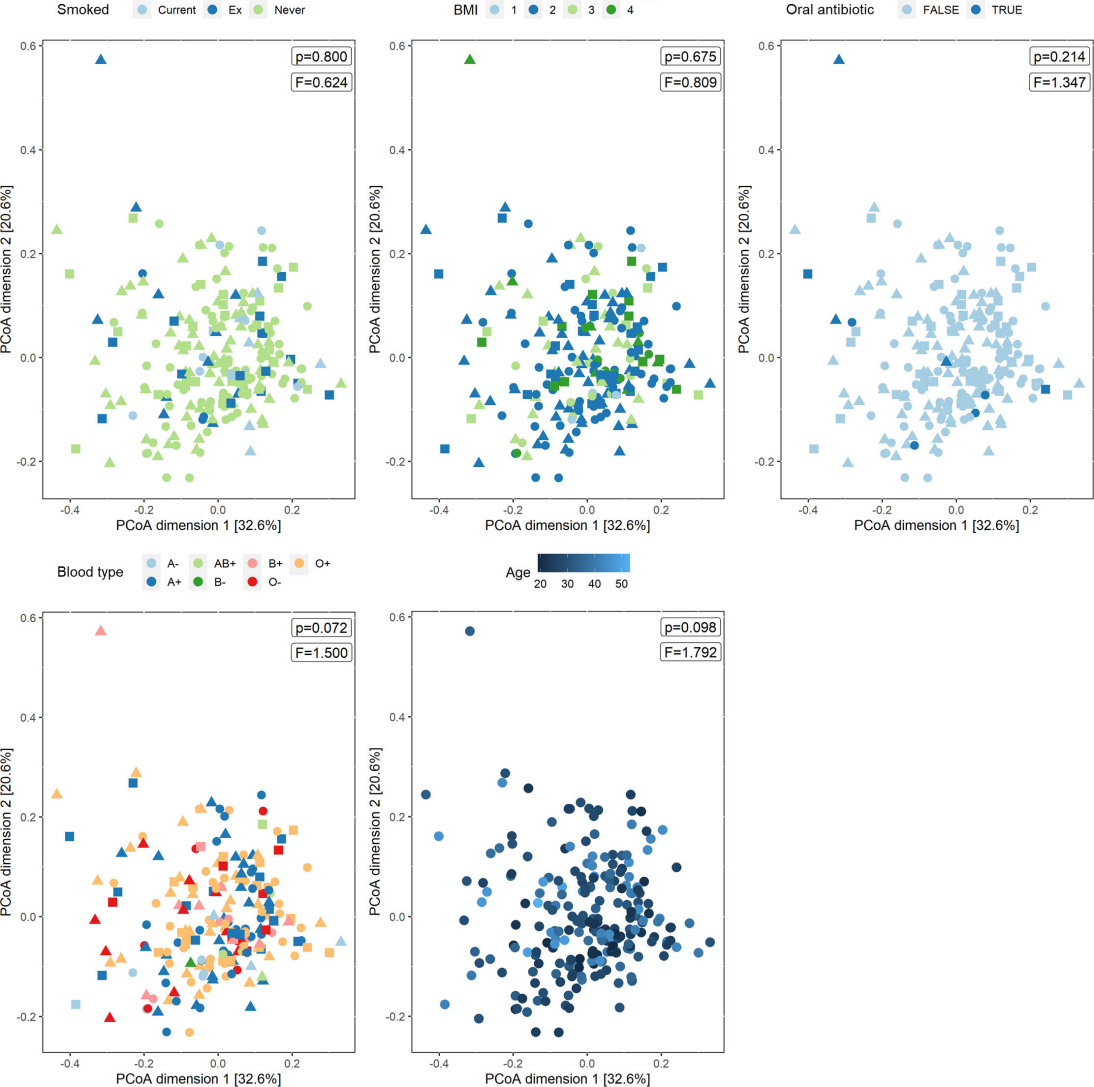
The influence of host-related variables on the beta-diversity of the salivary microbiome. PCoA plots to visualize potential correlations with several variables. Active smoker, BMI, recent oral antibiotic use, blood type, kissing, and age were investigated in relation to the bacterial profiles in the saliva. *P*-values (based on PERMANOVA tests) are shown for all tested covariates. For BMI, numbers indicate the different categories: 1 = BMI < 18.5 (underweight); 2 = 18.5 ≤ BMI ≤24.9 (healthy); 3 = 25.0 ≤ BMI ≤29.9 (overweight); and 4 = BMI ≥ 30.0 (obese). Shapes represent different age categories: circle = 18–28; triangle = 29–39; and square = 40–50.

We next investigated possible associations between metadata obtained through the survey on general host-related and lifestyle variables and the salivary microbiome. In order to explore the differences in overall microbial profiles (measured in terms of Bray-Curtis distance) associated with these variables, principal coordinates analysis (PCoA) was used (Fig. 3). Of interest, no significant impact of age (PERMANOVA, *P* = 0.098, *F* = 1.792) was observed. We also did not detect significant differences in microbial profiles associated with BMI (*P* = 0.675, *F* = 0.809) and smoking (*P* = 0.800, *F* = 0.624). We did observe a near-significant trend toward a change in diversity associated with blood type (Bray-Curtis distance, PERMANOVA, *P* = 0.072, *F* = 1.500).

**Fig 4:**
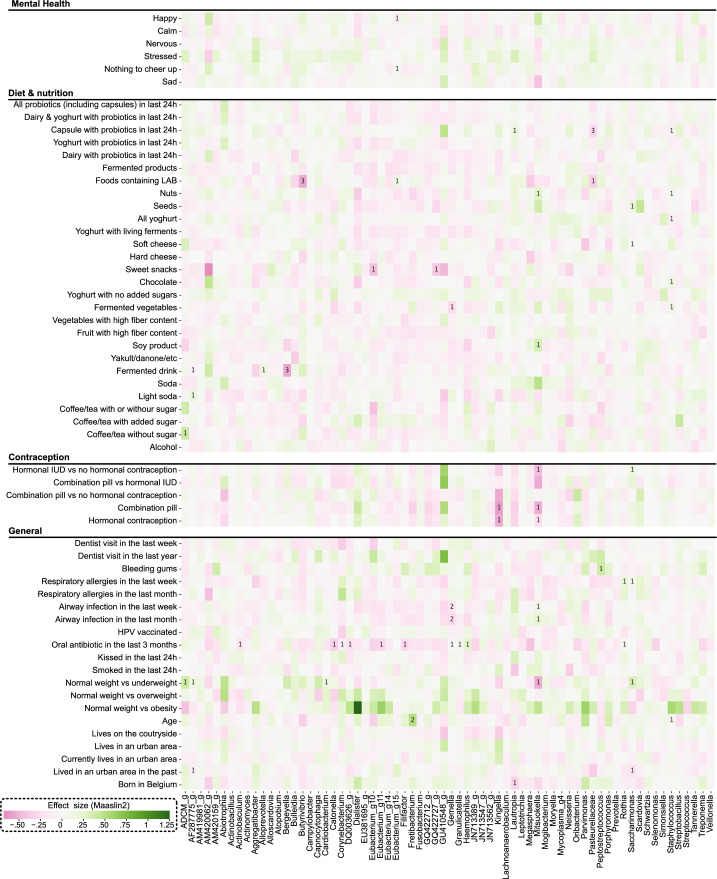
The effect of host and environmental variables as well as the consumption of certain food items up to 24 hours prior to sampling on the relative abundances of the 80 most abundant genera that were also present in at least 10% of samples. Numbers in the figure represent how many tools showed a significant association (linear regressions, FDR adjusted and using a threshold of 0.05). The number of samples for each question was the entire study population (*n* = 228). Except for the analysis regarding contraception, participants that used progesterone-only pill or vaginal ring as contraception method were removed (*n* = 224). Food-related questions were analyzed by correlating the number of portions with the abundance of certain genera.

When participants used oral antibiotics in the last three months (3.95%), no significant difference in microbial profiles was observed based on Bray-Curtis distance (*P* = 0.214, *F* = 1.347). However, a shift towards a lower alpha-diversity was found (Fig. S3). This was significant based on the genus richness (*P* = 0.025) but not significant based on Inverse Simpson Index (*P* = 0.23), indicating that the evenness of the microbial community was not affected. When the women used antibiotics in the last three months, we observed a significantly lower relative abundance at the genus level of *Actinobacillus*, *Catonella*, *Corynebacterium*, DQ003626_g, *Eubacterium*_g11, and *Filifactor* and higher relative abundance of *Gemella*, *Granulicatella*, *Haemophilus,* and *Rothia* based on one or two tools (associations on ASV level are shown in Fig. S4). Also for other host covariates analyzed, we assessed associations with specific taxa. An increase in relative abundance of the genera *Fretibacterium* and *Staphylococcus* and the ASVs *Gemella* ASV one and *Lachnoanaerobaculum* ASV two was observed with increasing age (Fig. 4; Fig. S4). Moreover, less *Mitsuokella* and more ADCM_g, AF287775_g, *Cardiobacterium,* and *Saccharimonas* were noted in participants with a normal weight compared to underweight (BMI <18.5) based on one tool.

We also investigated the association of other health-related factors, such as respiratory allergies/infections and oral health, with the salivary microbiome. Both oral and respiratory health were found to influence the abundance of certain taxa in the microbial community. More specifically, participants who suffered from bleeding gums in the week prior to sampling (10.96% of participants) appeared to have a higher abundance of the *Peptostreptococcus* genus in their salivary microbiome (Fig. 4). Having an airway infection in the last week or month resulted in a lower abundance of *Gemella* spp. and a higher abundance of *Mitsuokella* spp. (Fig. 4).

### Specific food components have a short-term impact on the salivary microbiome

Since food is frequently in direct contact with the salivary microbiome, we analyzed the effects of specific food items on the relative abundances of the most abundant taxa in more detail, based on the data on food items consumed during the 24 or 48 hours prior to sampling. In addition, we checked whether being pescatarian/vegetarian/omnivore had any impact, but no major associations with the microbial composition were observed (Fig. S5A). However, looking at specific genera/taxa, we detected several associations or by at least one multivariate tool. In Fig. 4, the short-term influences of portions of recently consumed food items (last 48 hours) on the most abundant genera are shown. Of interest, consumption of bacteria-containing products such as fermented foods and yoghurt was associated with changes in the abundance of several taxa. For example, consuming more fermented drinks was associated with a decrease in the abundance of the genera *Bergeyella* and AF287775_g and increase in the abundance of *Alloprevotella*. Consuming more foods containing lactic acid bacteria (LAB), including olives, kimchi and sauerkraut, was associated with a decrease *Butyrivibrio* and *Pasteurellaceae*. Consuming more yoghurt resulted in a lower abundance of *Staphylococcus* spp. The associations at ASV level are shown in Fig. S5 and in Table S2.

### Subtle associations of mental health status and the salivary microbiome

**Fig 5:**
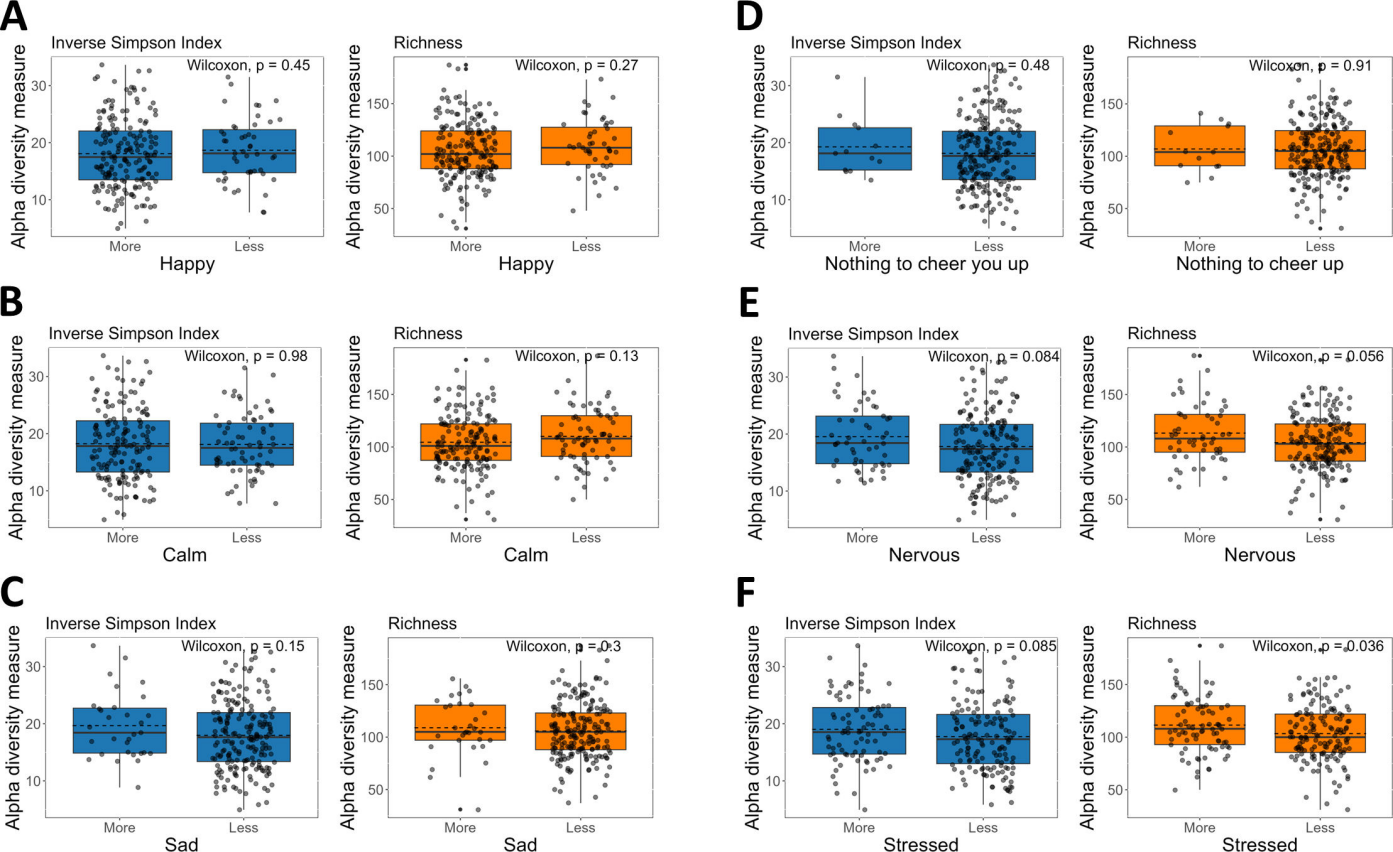
Influence of mental health on the alpha-diversity of the salivary microbiome based on the Inverse Simpson (left, blue) and richness (right, orange). *P*-values for diversity analysis are given in each figure (based on the Wilcoxon test). Analysis was performed on ASV level.

Finally, since the citizen-science project was set up during the first months of the COVID-19 pandemic, we also explored associations with mental health and stress of the participants. Although 79% of participants indicated to be more happy, almost one out of four participants indicated to be more nervous at the time of filling in the survey and 35% noted to be more stressed (Table 1). The association of different emotions with changes in alpha- (Fig. 5) and beta-diversity (Fig. S5B) of the microbial composition was analyzed. First, an analysis was done at the genus level, pointing towards a higher alpha-diversity in women who indicated to be more nervous or stressed was found (Fig. 5E and F). *P*-values of 0.056 and 0.036 for feeling more nervous and stressed, respectively, were calculated based on richness. No clear difference was observed for the Inverse Simpson index (Fig. 5) and beta-diversity (Fig. S5B) (Fig. S6) or the relative abundance of the most abundant taxa/genera (Fig. 4). This indicates that mental health is possibly influenced by the microbial community as a whole, indicated by the altered alpha-diversity of the salivary microbiome when feeling more stressed but not by specific genera or taxa.

## DISCUSSION

The Isala citizen science project is focused on mapping the microbiome of healthy women. The first phase of Isala was focused on the vaginal microbiome (20) but also other human body sites such as the oral cavity and skin were sampled. Here, employing the large database created during Isala, we were able to analyze the microbial communities of saliva from a cohort of generally healthy Belgian women and investigate the potential associations of metadata with the abundance of bacterial taxa as well as alpha- and beta-diversity. The first finding was the highly preserved microbial composition of the salivary microbiome across all female individuals participating. No separate clusters were defined within this Western-European female cohort. Previous studies did define two (35, 36), three (37), and even five (38) different clusters in the salivary microbiome profiles independent of gender. Nevertheless, these studies identified the same dominant genera as we observed here. De Filippis et al. (37) identified 12 genera present in 98% of saliva samples of healthy Italian citizens independent of gender (*n* = 161, age 18–55, 42% male), namely *Streptococcus, Veillonella, Gemella, Granulicatella, Neisseria, Prevotella, Rothia, Fusobacterium, Porphyromonas, Leptotrichia, Haemophilus,* and *Actinomyces* based on V1–V3 region 16S rRNA gene amplicon sequencing. The first eight were also identified as being the core salivary microbiome in healthy US citizens (*n* = 107, age 8–26) independent of gender (39). Similar to De Filippis et al. (37), our data revealed 12 genera to be present in more than 98% of all saliva samples from women but with subtle differences namely *Actinobacillus, Alloprevotella,* and *Campylobacter* found in our samples instead of *Rothia*, *Porphyromonas,* and *Haemophilus*. The fact that there is a clear similarity between the most prevalent genera in our data set and the ones identified as core microbiome by others (37, 39), supports the presence of highly prevalent microbial taxa among healthy individuals (37).

Within this salivary microbial community and its apparent stability in healthy women, we next aimed to investigate associations of relevant host-related and lifestyle variables. A primary interest of the Isala study was the association with intimate partnership, as it is known that human beings exchange vast amounts of microbes with their intimate partners at different body sites (vagina, skin, and saliva, the latter studied here). A previous study found clear correlations between the similarity indices of the salivary microbiota of couples and self-reported kissing frequencies (5). This study particularly reported the short-term impact of microbial exchange, with an average bacterial transfer of 80 million bacteria per intimate kiss of 10 seconds (5). In our study, we investigated the alpha- and beta-diversity of the salivary microbiome of women who had no, one, or multiple steady intimate partners. In addition to this information, our participants also indicated if they kissed someone in the 24 hours prior to taking the saliva sample, which we hypothesized to result in a more diverse salivary microbiome. However, no association with both variables was observed in our data. This is in line with the fact that the previous results of Kort and colleagues indicate that the effect of an intimate kiss on the microbial community is greatest within a period of 1 hour 45 min after the kiss (5). Our data now adds that women with more than one intimate partner do not have a more diverse salivary microbiome independent of sex and/or heritance.

A second major factor of interest was hormonal contraception because it is one of the most prescribed medications for women of reproductive age (19). We did not observe an association between the type of contraception and beta-diversity and only small associations with specific taxa in the saliva microbiome such as the genera *Kingella* and *Mitsuokella*. A near-significant lower alpha-diversity in participants who use a hormonal IUD based on Inverse Simpson (*P* = 0.062) was observed. To the best of our knowledge, only two recent studies have investigated the influence of the hormonal contraception on the salivary microbiome using next-generation sequencing techniques (40, 41). A study of 103 women of reproductive age with a regular menstrual cycle in Denmark (22–29 years old) did not show an impact of contraceptive use but they had a smaller number of participants with a hormonal IUD (18.45%) compared to our study (32.02%) (40). In another study [also in Denmark, partially the same study group as (40)] that included 160 women (18–40 years old) with a similar ratio of IUD use as ours, also no impact of contraceptive use was observed (41).

In addition to these contraception-related and intimate partnership-related factors, we did not observe a clear association between age, blood type, antibiotic use, BMI or smoking, and overall salivary microbiome composition (beta-diversity). However, we did find some potential associations between the relative abundances of specific genera and/or ASVs and oral antibiotic use, low BMI, oral health (bleeding gums and dentist visit), and age. The biggest potential influence found was the use of oral antibiotics, which significantly lowered the alpha-diversity based on bacterial richness. This is in line with previous reports that describe the influence of antimicrobials on the saliva microbiota in children (17, 18). Of interest, in our data presented here, we observed a lower relative abundance of *Actinobacillus*, *Catonella, Corynebacterium,* DQ003626_G, *Eubacterium_g11,* and *Filifactor* and a higher relative abundance of *Gemella, Granulicatella, Haemophilus,* and *Rothia* after antibiotic use. Zaura et al. (42) investigated the effect of several antibiotics on the gut and salivary microbiome, demonstrating minor changes in beta-diversity and influences on individual microbial taxa such as a decrease in *Haemophilus, Actinobacillus, Porphyromonas,* and *Parvimonas* by ciprofloxacin. These and our own results clearly demonstrate the systemic effects of antibiotics on the human microbiome, in agreement with data for other human habitats [reviewed in (43)]. Of note, only 3.95% of participants reported to have taken oral antibiotics in the last 3 months, so that our study was not powered enough to detect subtle differences.

Regarding oral health, factors such as bleeding gums and dentist visits were shown to be associated with the oral microbiome, in line with previous research (44 – 46). Our data particularly indicated a higher relative abundance of *Peptostreptococcus* genus in participants who suffered from bleeding gums. Lundmark et al. (44) previously linked periodontitis with a higher abundance of. for example. *Streptococcus mitis/parasanguinis, Parvimonas micra, Filifactor alocis,* and *Prevotella* sp., while Damgaard et al. (46) found a higher abundance of *P. gingivalis, P. aurantiaca, P. endodontalis.* and Ko et al. (45) of genera. including *Peptostreptococcus, Treponema, Parvimonas, Alloprevotella.* and *Filifactor.* in patients with periodontitis. The unhealthy habit of smoking is another major factor in oral and respiratory health. Several studies described changes in the abundance of several genera as well as lower diversity in smokers (13 – 15, 40, 47). Remarkably, we did not observe a clear association with smoking. However, these previous studies had a greater smoking cohort compared to our study, which only included 7.02% active smokers. Related to respiratory health, more specifically having an airway infection in the last week or month, was associated with a lower abundance of *Gemella* spp. and a higher abundance of *Mitsuokella* spp in our cohort. Several studies have shown an association of respiratory infection and a change in the relative abundance of certain bacterial taxa in saliva (48 – 50). For example, Kim et al. have indicated a depletion of *Bifidobacterium*, *Lactobacillus.* and *Solobacterium* in patients with severe COVID-19 (48). However, more dedicated research is needed to look more into this association.

Consequently, we also analyzed the short-term influences of portions of different food items on the salivary microbial composition. We mainly observed changes in the relative abundance of some taxa when consuming more food that contains bacteria such as fermented foods or yoghurt. For example, a decrease in *Staphylococcus* spp. was associated with the consumption of more yoghurt . Additionally, consuming more foods containing LAB resulted in an increase of *Alloprevotella* and a decrease in *Pasteurellaceae,* respectively. Consumption of fermented milk or probiotic yoghurt containing *Streptococci* and *Lactobacillaceae* has been previously shown to result in a (short) term increase of these genera (51). The study of Arweiler et al. (52) showed that the use of several probiotic products (Symbioflor 1, Actimel Classic. and InfectoDiarrstop) resulted in a reduction of *Streptococcus* even after washout, but no changes in probiotic bacteria (*Enterococcus faecalis, Lacticaseibacillus casei,* or *Lacticaseibacillus rhamnosus*) were observed (52).

Finally, we investigated possible associations between mental health and the salivary microbiome. Previous studies had already indicated that depression and anxiety could impact on the salivary microbiome, on diversity (53), and/or on the microbial composition (53, 54). Our study during the stressful COVID lock-down period provided a unique opportunity to investigate more subtle effects on mental health. We indeed observed changes in alpha-diversity for higher levels of being stressed and nervous (observed diversity, *P*-values of 0.036 and 0.058, respectively) but not for specific genera or taxa. Changes in the oral microbiome due to stress/nervousness/depression/anxiety could be due to higher levels of cortisol in saliva as stated by Simpson et al. (54). However, previous research showed that people with depression are more likely to smoke and often suffer from worse oral health (53) which could also result in changes in the oral microbiome. Taken together, further investigation is required to elucidate the effect of mental health on microbial composition in adults.

Regarding study limitations, it is important to highlight that our study used 16S amplicon sequencing (V4 region), which has advantages such as broad taxonomic coverage of bacteria, low price, and small proportions of amplified host DNA (mitochondria). The V4 region specifically allows for almost full overlap between forward and reverse reads, which enables more stringent quality control, but it also comes with bias for some genera such as *Streptococcus* for which other regions such as V1–V3 are more suitable. In addition, 16S sequencing does not allow for the sequencing of fungi, while recent research indicated the importance of the complex interactions of bacteria and fungi in saliva (55). Therefore, approaches such as metagenomic shotgun sequencing are of interest for follow up work to identify the salivary microbiome at a lower taxonomic and more functional level. However, hereto, further optimization and generalization of the processing of saliva samples for metagenomic sequencing is needed because our previous work (21) has revealed the presence of a taxonomic bias depending on DNA extraction method employed. Another limitation of our work was the citizen science-based approach with a strong focus on women’s health and possible associated bias because we aimed to investigate associations with vagina, skin, and saliva microbiome. For example, our participants identified themselves as healthy, and the sample size was too small to observe significant influences for many factors such as for smoking. However, by controlling the power for our main factors of interest and giving clear sampling instructions, reducing the recall time as much as possible and anonymizing the study, we have maximized the advantages and power of citizen science to reach the general population while minimizing bias as much as possible. Finally, although our study contained 246 women due to the large variation in the composition of the oral microbiome, participants’ lifestyles and the large number of tests performed, we may lack power to detect subtle effects. A negative result here does therefore not necessarily indicate that there is no effect of these factors on the salivary microbiome, rather we should expect that the effects are likely to be subtle.

### Conclusion

This citizen’s science study established the salivary microbiome of healthy women in Belgium. The salivary microbiome is characterized by highly prevalent microbial taxa among this set of female individuals. Additionally, using the large metadata set, we found potential influences of biological (e.g., age and BMI) as well as lifestyle factors (e.g., antibiotic use, oral health, and mental health) on the lower abundant genera and/or ASVs.

## Data Availability

Sequencing data are available at the European Nucleotide Archive (ENA) with the accession number PRJEB55042.

## SUPPLEMENTAL MATERIAL

Supplemental figures mbio.00300-23-s0001.pdf Fig. S1 to S6.

Supplemental File S1 mbio.00300-23-s0002.docx The online questionnaire used in this citizen science project. These questions provided information on general health, hygiene and food intake.

Table S1 mbio.00300-23-s0003.docx
**Demographics of non-responders.** This information is based on all participants that did not fill in the questionnaire accompanying the sample and/or did not send in a saliva sample.

Table S2 mbio.00300-23-s0004.csv
**An overview of the effect of host and environmental variables as well as the consumption of certain food items 24h prior to sampling on the relative abundances of the 80 most abundant ASVs that were also present in at least 10% of samples.** All results are presented with the analyzed population, effect size, uncorrected and corrected p-value. The number of samples for each question was the entire study population (*n* = 228). Except for the analysis regarding contraception, participants that used progesterone-only pill or vaginal ring as contraception method were removed (*n* = 224). Food-related questions were analyzed by correlating the number of portions with the abundance of certain ASVs.

Table S3 mbio.00300-23-s0005.csv
**An overview of the effect of host and environmental variables as well as the consumption of certain food items 24h prior to sampling on the relative abundances of the 80 most abundant genera that were also present in at least 10% of samples.** All results are presented with the analyzed population, effect size, uncorrected and corrected p-value. The number of samples for each question was the entire study population (*n* = 228). Except for the analysis regarding contraception, participants that used progesterone-only pill or vaginal ring as contraception method were removed (*n* = 224). Food-related questions were analyzed by correlating the number of portions with the abundance of certain genera.

ASM does not own the copyrights to Supplemental Material that may be linked to, or accessed through, an article. The authors have granted ASM a non-exclusive, world-wide license to publish the Supplemental Material files. Please contact the corresponding author directly for reuse.
