# Supplementary material for: Salivary microbiome of healthy women of reproductive age
Source: mBio. 2023 Sep 1;14(5):e00300-23. doi: 10.1128/mbio.00300-23 (PMC10653790; doi:10.1128/mbio.00300-23)
Supplement: Supplemental figures — Fig. S1 to S6. [file mbio.00300-23-s0001.pdf]

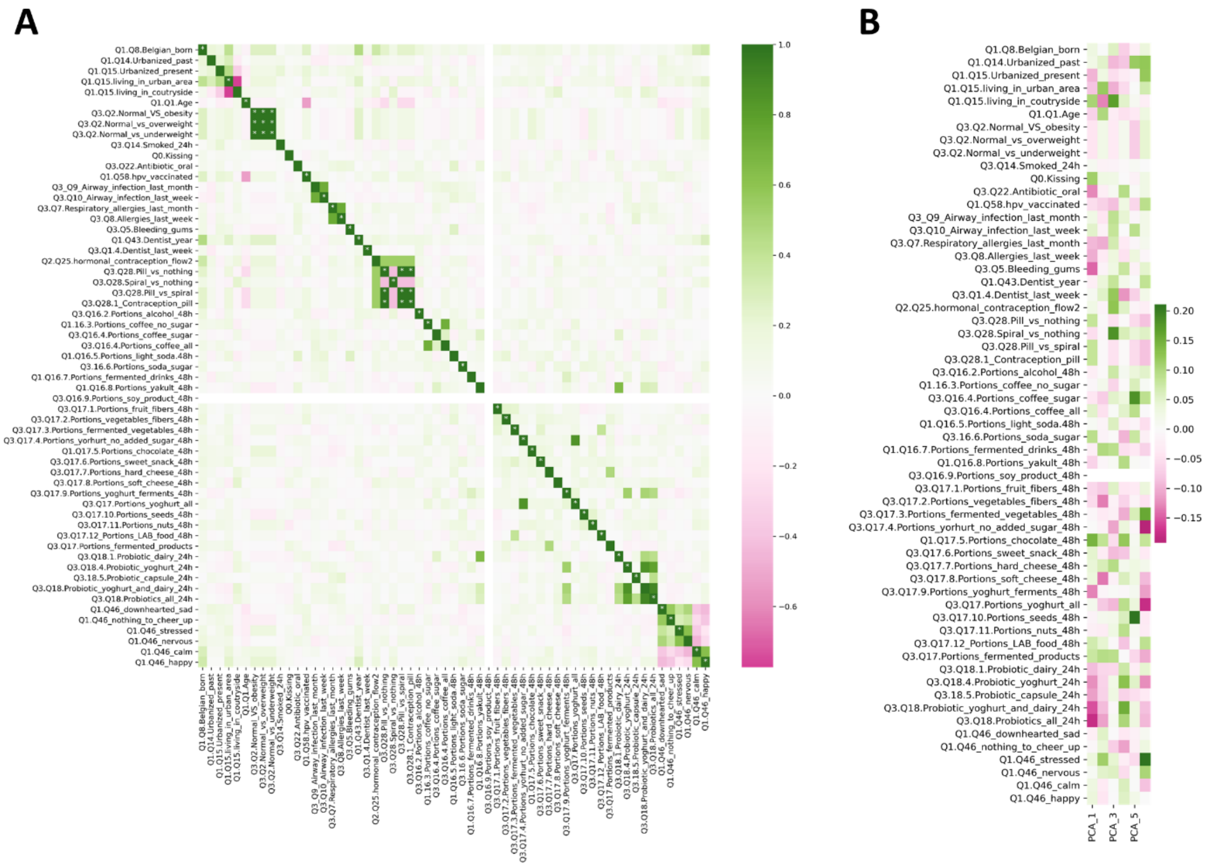

Figure S1: Statistical analysis of the confounding factors. (A) Investigation of how covariates are confounded to one another based on Pearson correlation. (B) Association of the covariates with the first six principle components of the Bray-Curtis beta-diversity matrix of the abundance data based on simple linear regression.

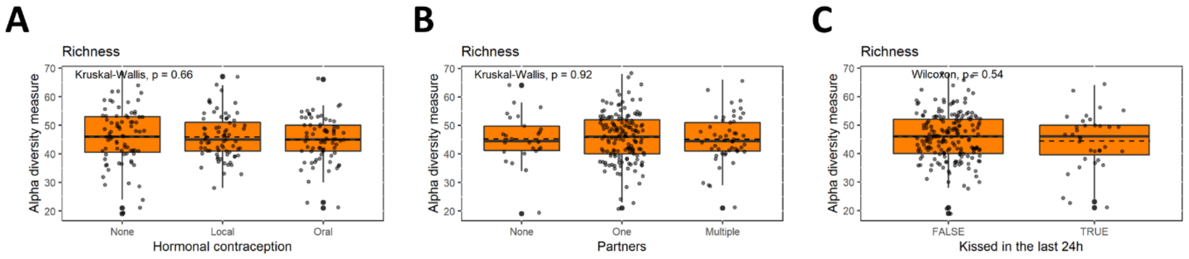

Figure S2: Analysis of the influence of (A) hormonal contraception, (B) number of partners and (C) kissed in the last 24 hours on the alpha-diversity of the salivary microbiome in women. P-values are given for each analysis, calculated based on Kruskal-Wallis or Wilcoxon.

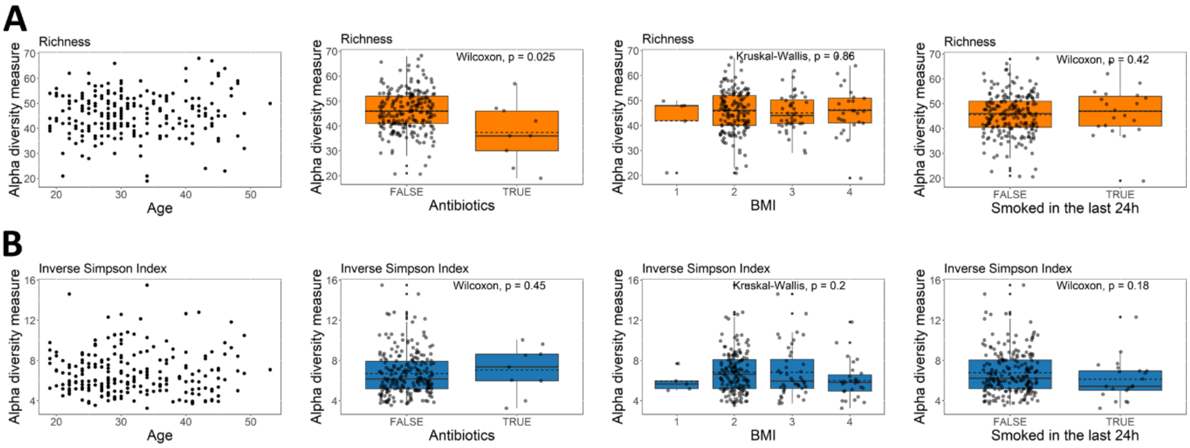

Figure S3: Statistical analysis of the association of host-related variables (age, antibiotics, BMI and smoking) with the alpha-diversity of the salivary microbiome based on (A) richness and (B) Inverse Simpson Index. For BMI, numbers indicate the different categories: 1 = BMI < 18.5 (underweight); 2 = 18.5 ≤ BMI ≤ 24.9 (healthy); 3 = 25.0 ≤ BMI ≤ 29.9 (overweight); 4 = BMI > 30.0 (obese). P-values are given for each analysis, calculated based on Kruskal-Willis or Wilcoxon.

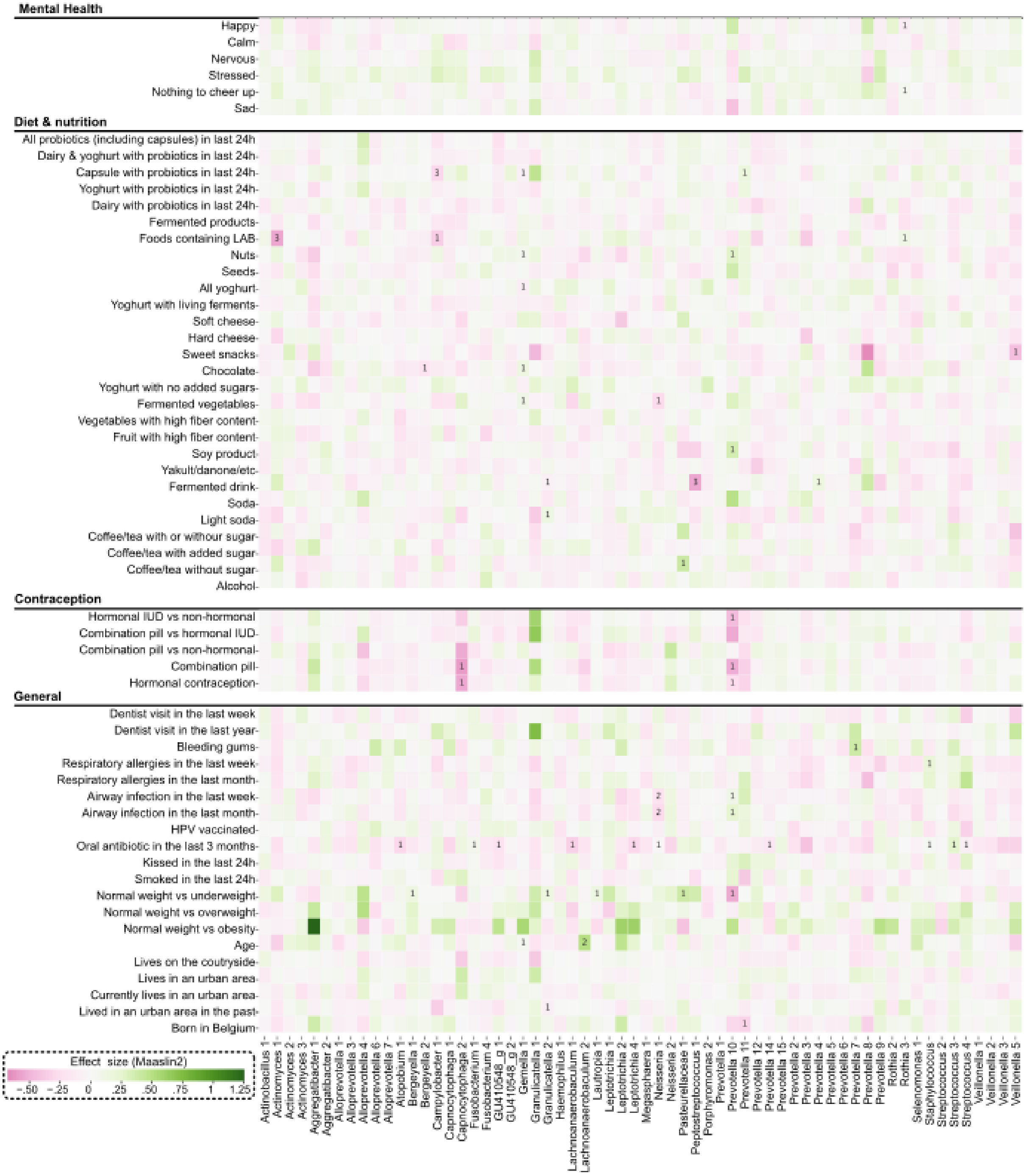

Figure S4: The effect of host and environmental variables as well as the consumption of certain food items 24h prior to sampling on the relative abundances of the 80 most abundant ASVs that were also present in at least 10% of samples. Asterisks represent significant associations (linear regressions, FDR adjusted and using a threshold of 0.05). The number of samples for each question was the entire study population (n = 228). Except for the analysis regarding contraception, participants that used progesterone-only pill or vaginal ring as contraception method were removed (n = 224). Food-related questions were analyzed by correlating the number of portions with the abundance of certain ASVs.

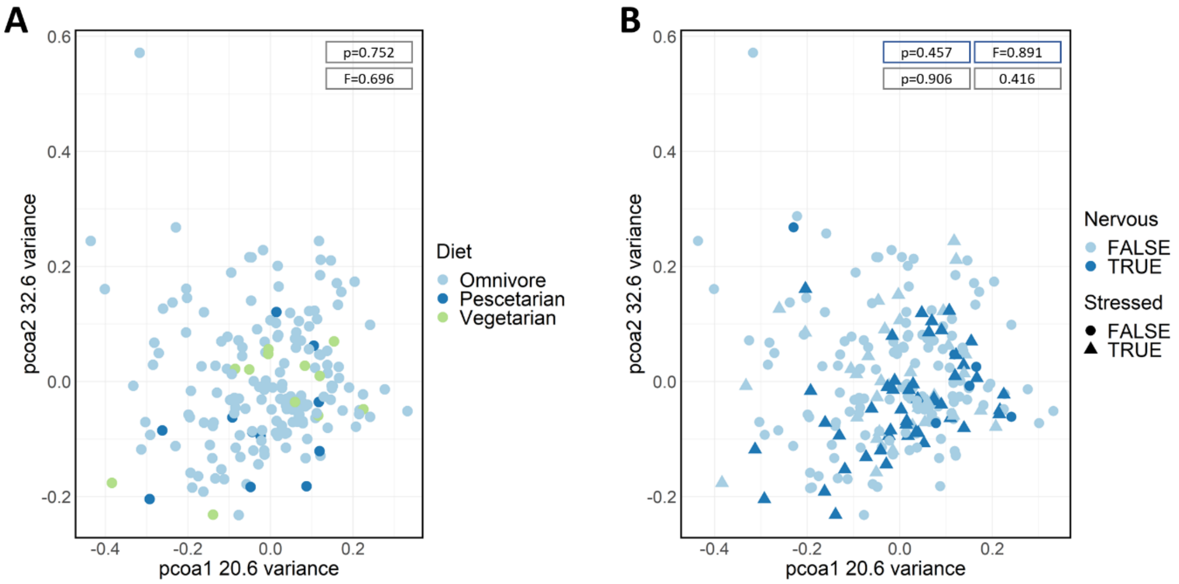

Figure S5: The association of diet and mental health on the beta-diversity of the salivary microbiome. PCoA plot distributing the samples according to beta-diversity (Bray-Curtis distance). (A) Samples are colored by different diets, i.e. omnivore, pescatarian or vegetarian. (B) Samples are colored by being nervous or not and shapes indicate being stressed or not. P-values (based on adonis) are indicated.

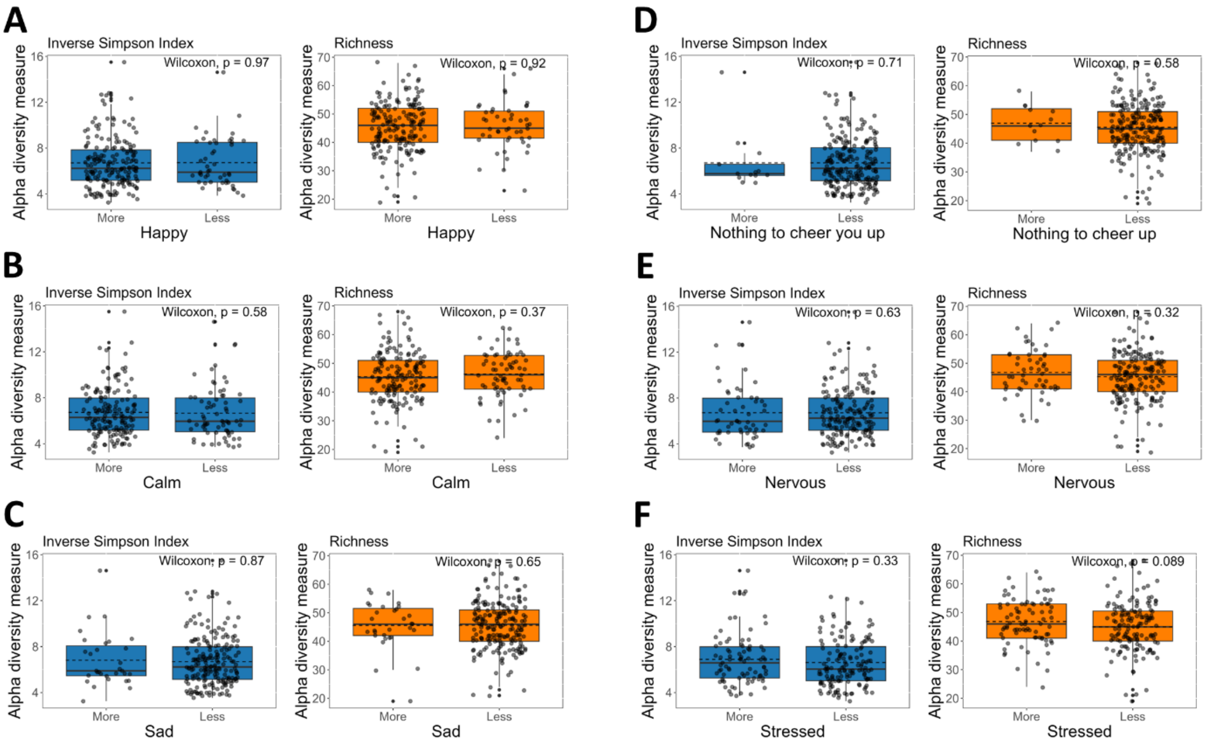

Figure S6: Influence of mental health on the alpha-diversity of the salivary microbiome based on the Inverse Simpson (left, blue) and richness (right, orange). P-values for diversity analysis are given in each figure (based on the Wilcoxon test). Analysis was done on genus level.
