# Supplementary material for: Salivary microbiome of healthy women of reproductive age
Source: mBio. 2023 Sep 1;14(5):e00300-23. doi: 10.1128/mbio.00300-23 (PMC10653790; doi:10.1128/mbio.00300-23)
Supplement: Supplemental File S1 — The online questionnaire used in this citizen science project. These questions provided information on general health, hygiene and food intake. [file mbio.00300-23-s0002.docx]

**QUESTIONNAIRE**

Version 4 - April 14, 2020

**Integrated genetic and functional analysis of the female microbiome in a Flemish cohort**

Completing the questionnaire takes about 20 minutes. All questions in the questionnaire probe factors that affect the bacteria on your body and are important to correctly interpret Isala's results. Therefore, we ask you to complete all the questions. The survey is designed so that your personal data cannot be directly linked to your answers to ensure your privacy. If it is difficult to complete this questionnaire, you can always contact us at isala@uantwerpen.be

**General**

Your personal study number^[[1]](#footnote-2)^ : .........................................

Gender:

- Woman
- Man
- Transman: since when? ... (year)
- Trans woman: since when? .... (year)
- Otherwise

Age: .........years

Length: ............... cm

Weight: .... ........kg

This is the best way to describe my current relationship status:

- Permanent partner
- Permanent partner, not cohabiting
- Changing partners
- Bachelor

My partner(s) is/are....

- Man
- Woman
- Both
- No

This is what my family looks like:

- Living with partner or married
  - If yes, how long have you lived together (circle the appropriate answer)?
    - Less than 3 months
    - Between 3 months and 1 year
    - Between 1 year and 5 years
    - Between 5 years and 10 years
    - More than 10 years
- Living with friends or other roommates
  - If yes, how long have you lived together (circle the appropriate answer)?
    - Less than 3 months
    - Between 3 months and 1 year
    - Between 1 year and 5 years
    - Between 5 years and 10 years
    - More than 10 years
- Living with relatives
  - If yes, how long have you lived together (circle the appropriate answer)?
    - Less than 3 months
    - Between 3 months and 1 year
    - Between 1 year and 5 years
    - Between 5 years and 10 years
    - More than 10 years
- Single parent
- Single parent with co-parenting
- Living alone

If you are not living alone, how many people do you live with in the same house, yourself not included (even if not all the time)?........................

Do you have children?

- Biological children: how many?.............................
  - How did you give birth?
    - By cesarean section:..... times
    - Natural childbirth: ...... times
- Adopted children or foster children: how many?.............................
- Plus children (= stepchildren) : how many?............................
- No children

How would you describe the area where you grew up? This question is about the place where you spent most of your childhood. When in doubt, choose the place where you first spent a long time.

- City Center
- Village center
- Residential residential area
- Busy job
- Rural area
- Green zone/recreation area/....
- Industrial Zone

How would you describe the area where you live now?

- City Center
- Village center
- Residential residential area
- Busy job
- Rural area
- Green zone/recreation area/....
- Industrial Zone

Do you find yourself daily in an environment with.....

- Lots of greenery
- Lots of greenery
- Fair amount of greenery
- Little greenery
- Very little greenery
- No green

Were you born in Belgium?

- Yes
- No
  - What country were you born in?...........................
  - How old were you when you came to Belgium?............................
  - Do you live permanently (more than 6 months) in Belgium? ...................

What country(ies) did you live in until the age of three? .........................

What nationality(ies) does your birth mother have?.................

What nationality(ies) does your biological father have? ....................

Are there other cultures/countries you also identify with besides Belgium?

- Yes, which one?...........................
- No

Were you abroad (for at least three consecutive days) in the past three months?

- Yes, to which country or countries? ...................................................
- No

What is the highest degree you earned?

- Elementary school
- Secondary education: ASO
- Secondary education: KSO
- Secondary education: TSO
- Secondary education: BSO
- College: bachelor's degree
- University: bachelor's/master's degree (candidate/licensee)
- Postgraduate: doctor
- Other: ..........................
- No

What industry do you work in?

- Hospitality and tourism
- Education
- Event agencies, audiovisual and film sector
- Social economy
- Consultancy and other services to companies
- Real estate and facility services
- Food, tobacco
- Transportation, logistics
- Textiles, clothing
- Industry (general)
- Government and public administrations
- Socio-cultural sector, sports, youth work, ...
- Agriculture, horticulture, forestry, fisheries
- Computer services and companies
- HR and employment agencies
- Wood and furniture industry
- Graphic, paper and board industry
- Health services and institutions
- Energy, water
- Chemistry, metal
- Construction
- Banking, insurance, mail, telecom
- Trade, rental
- Other: .............

Describe your work situation under normal circumstances:

|  | Daily | More than 3x/week | Weekly | Monthly | Rarely | Never |
| --- | --- | --- | --- | --- | --- | --- |
| Contact with other people |  |  |  |  |  |  |
| Contact with chemicals (e.g., chlorine, flame retardants, Teflon, acetone, disinfectant, lab equipment, etc.) |  |  |  |  |  |  |
| Physical contact with outdoor plants |  |  |  |  |  |  |
| Physical contact with indoor plants |  |  |  |  |  |  |

Do you have contact with animals while at work or at home?

- Yes,
  - At work, how many and which animals?..................................................
  - I have pets,
    - Indoors, how many and what animals?............................................
    - Outdoors, how many and what animals?............................................
- No

Indicate your work rhythm. If you combine several jobs, add everything up (multiple answers are possible)

- Full-time
- Part-time: .......%
- Long-term sick leave (more than three months)
- Unemployed
- (Early) retirement
- Student
- Volunteer
- Other: ..............

I usually work (multiple answers possible)

- During the day
- overnight
- In shifts:
- Early shift
- Late shift
- Nights
- Other: ..................

Do you smoke?

- Yes, approximately how many cigarettes do you smoke per day?...................................
  - How long have you been smoking?............................ (years/months)
- No, ex-smoker
  - Approximately how many cigarettes did you smoke per day in the past? ...........
  - How many years/months ago was this? ...............................
  - How long have you smoked?..................... (years/months)
- No, never smoked

Do you use drugs (excluding alcohol)? Please also indicate which ones. This information will be processed pseudonymously. We have the utmost respect for you and for your privacy.

- Yes, what drugs?...................................
  - What is the frequency of your current drug use?
    - Several times a day
    - Daily
    - More than 3x/week
    - Weekly
    - Monthly
    - Rarely
  - How long have you been using drugs? ............(years/months)
- No, ex-drug user: what drugs?................
  - What was the frequency of your drug use at the time?
    - Several times a day
    - Daily
    - More than 3x/week
    - Weekly
    - Monthly
    - Rarely
  - How many years/months ago was this? ...............................
  - How long have you been using drugs? ....................(years/months)
- No, never used drugs

On average, how many hours do you sleep per night?

- On weekdays:..........h.
- On days off: ..................h.

How many hours a week do you exercise (multiple answers possible)?

- I don't do sports
- Moderate physical activity: ........... h.
- Intense physical activity: ............. h

What sports do you play? ................................

On average, how many minutes/hours a day do you spend outside ?

During the week..............................

On weekends..................

**Diet**

Do you take probiotics or "good bacteria" (both eating and drinking) (multiple answers possible)?

- In dairy products such as Yakult, Actimel?
  - Yes
    - How often? several times a day/ daily/ more than 3 times a week/ weekly/ monthly/ rarely/ never
    - Which products? ........................................................................
    - Have you used such products in the last month? ...........................
  - No
- In capsules such as Enterol, Probactiol or other similar dietary supplements from pharmacies or drug stores?
  - Yes
    - How often? several times a day/ daily/ more than 3 times a week/ weekly/ monthly/ rarely/ never
    - Which products? ........................................................................
    - Have you used any such products in the past month? ...........................
- In yogurt?
  - Yes
    - How often? several times a day/ daily/ more than 3 times a week/ weekly/ monthly/ rarely/ never
    - Which products? ........................................................................
    - Have you used any such products in the past month? ...........................
  - No

How regularly did you eat or drink the items listed below in the past 3 months?

|  | Several times a day | Daily | More than 3x/week | Weekly | Monthly | Rarely | Never |
| --- | --- | --- | --- | --- | --- | --- | --- |
| Dairy products (cheese, milk, yogurt, buttermilk, etc.) |  |  |  |  |  |  |  |
| Fermented foods (olives, salami, sauerkraut, sourdough bread, pickles, ... ) |  |  |  |  |  |  |  |
| Alcohol |  |  |  |  |  |  |  |
| Meat |  |  |  |  |  |  |  |
| Animal products (eggs, cheese, gelatin,...) |  |  |  |  |  |  |  |
| Fish |  |  |  |  |  |  |  |
| Sweet drinks with sugar (e.g., soda, fruit juice) |  |  |  |  |  |  |  |
| Sugar-free sweet drinks (e.g., light sodas) |  |  |  |  |  |  |  |
| Fruit |  |  |  |  |  |  |  |
| Vegetables |  |  |  |  |  |  |  |

When you drink alcohol, what do you usually drink? (multiple answers possible)

- Wine and/or sparkling wine
- Beer
- Spirits
- I don't drink alcohol
- Other:................

Do you take any vitamins, minerals or other supplements? If so, please also fill in which ones?

- Yes, regularly, throughout the year: which?...............
- Yes sometimes, during a certain period of time: which one?...............
- No

**General health**

How were you born?

- By cesarean section
- With a vaginal delivery
- Don't know

What is your blood type (Circle your rhesus factor if you know it)?

- A : + / -
- B: + / -
- AB : + / -
- O : + / -
- Don't know

How do you assess your general state of health?

- Very good
- Good
- Fair
- Bad
- Very bad

Do you have any of the conditions listed below? (Please state the condition)

| System/all over your body  E.g. rheumatism, arthritis, MS, ... |  |
| --- | --- |
| Skin  Vb. Psoriasis, acne, ... |  |
| Heart/blood vessels  Eg. endocarditis, aneurysm, ... |  |
| Stomach/intestines  Eg. Crohn's disease, colitis, ... |  |
| Airways  E.g. asthma, allergies, ... |  |
| Eyes  Vb. Glaucoma, cataracts,... |  |
| Nose-throat-ear  E.g. chronic sinusitis |  |
| Hormonal (e.g., thyroid) or diabetes (type 1/type 2) |  |
| Reproductive System  Vb. Endometriosis |  |
| Blood  Vb. Anemia, ... |  |
| Cancer Leukemia, breast cancer, ... | Sub-question: How many years/months ago was your diagnosis?........... |
| Other |  |

Have you ever received any of the following diagnoses?

- Allergy
  - Asthma
    - How many episodes of wheezing did you have in the last 12 months that were not related to a cold?
      - No
      - 1-3
      - 4-12
      - Over 12
  - Hay fever (pollen allergy)
  - House dust mite allergy
  - Animal allergy, which one? ............................
  - Other inhalant allergy: ......................
    - How many episodes of runny nose and/or itchy eyes did you have in the last 12 months, related to an inhalation allergy (pollen, dust mites, animals, ...)? ....
      - Less than 4 days/week or less than 4 weeks/year, and NOT disruptive to daily activities
      - Less than 4 days/week or less than 4 weeks/year, and disruptive to daily activities
      - More than 4 days/week and more than 4 weeks/year, and NOT interfering with daily activities
      - More than 4 days/week and more than 4 weeks/year, and disruptive to daily activities
      - No
  - Eczema
    - How often do you have flare-ups of eczema?
      - Several times a day
      - Daily
      - Weekly
      - Monthly
      - Rarely
  - Food allergy to ......................
  - Other: ......................
- Eating Disorder
  - Anorexia nervosa
  - Bulimia nervosa
  - Binge Eating Disorder
  - Other: ..............................
- Other condition: ................................
- No

Are you lactose intolerant?

- Yes
- No

Do you have a gluten allergy?

- Yes
- No

Have you had any antibiotic or antimycotic^[[2]](#footnote-3)^ treatment in the past three months (also report vaginal antibiotics and antimycotics such as e.g. gynodaktarin please)?

- Yes, how often?..................
- No

When did your last antibiotic treatment take place (approximately)?

................................................

In the past three months, did you take prescription medication? If yes, can you indicate how many different products were involved?

| Name | Dose | Start date | Stop date | Reason |
| --- | --- | --- | --- | --- |
|  |  |  |  |  |
|  |  |  |  |  |
|  |  |  |  |  |
|  |  |  |  |  |

Have you been to the dentist in the last 12 months?

- Yes
- No

Have you experienced regular bleeding gums in the last 12 months?

- Yes
- No

Have you suffered from caries or cavities in the past 12 months?

- Yes
- No

How did you feel the past four weeks?

| Question | Ongoing | Mostly | Often | Sometimes | Rarely | Never |
| --- | --- | --- | --- | --- | --- | --- |
| Did you feel very nervous? |  |  |  |  |  |  |
| Were you so badly down that nothing could cheer you up? |  |  |  |  |  |  |
| Did you feel calm and peaceful? |  |  |  |  |  |  |
| Did you feel dejected and gloomy? |  |  |  |  |  |  |
| Did you feel happy? |  |  |  |  |  |  |
| Did you feel stressed? |  |  |  |  |  |  |

**Reproductive and sexual health**

Have you had one or more sexual partner(s) in the past three months?

- Yes, one permanent partner
- Yes, multiple permanent partners: how many?....................................
- Yes, changing partners : how many?.................................
- No

If yes, was this sexual contact with :

- Woman(s)
- Male(s)
- Female(s) and male(s )

What sexual acts have you performed in the past 3 months? (multiple answers possible)

- Manual sex
- Oral sex
- Vaginal penetration with
- Fingers
- Toys
- Other: ...........
- Anal sex
- Other: ...........

What sexual acts have you received in the past 3 months? (multiple answers possible)

- Manual sex
- Oral sex
- Vaginal penetration with
- Fingers
- Penis
- Toys
- Other: ...........
- Anal sex
- Other: ...........

Have you had vaginal sex in the past 3 months, and if so, how frequently?

- No
- One exceptional time
- About once a month
- Several times a month
- About once a week
- Several times a week
- Daily

Do you have vaginal sex during your monthly bleeding?

- Yes
- No
- Sometimes

Did you experience any difficulties in fulfilling your pregnancy wish at that time?

- Yes
  - Did you encounter any problems with this then?
    - No
    - Yes
      - If Yes, did you see your family doctor/gynecologist for this?
        - Yes
        - No
- No

Has a fertility program ever been initiated for you? (multiple answers are possible)

- Yes, because of my reduced fertility with known cause, namely .......
- Yes, because of reduced fertility in my partner
- Yes, because of unexplained reduced fertility
- Yes, because me and my female partner want(s) to become mothers
- Yes, because I want(d) to become a mother without a partner
- Yes, because of another reason .........................................
- No

If yes, what type(s) of fertility program(s) did you follow? (multiple answers are possible)

- Hormone therapy
- Artificial insemination
- In vitro fertilization (IVF)
- Intracytoplasmic sperm injection (ICSI)
- Other:.........................................

How many times have you gone through such a process? .................................

How many trajectories/cycles of these resulted in pregnancy? .................................

How many trajectories/cycles of these resulted in the birth of a child? ........................

How many times have you been pregnant?.....................................

In the table below, provide more explanation for each pregnancy

| 1^e^ | Was the pregnancy planned?   - Scheduled - Unscheduled   Did you carry the pregnancy to term (until at least 37 weeks of pregnancy)?   - Yes - No, premature birth at ... weeks. - No aborted, spontaneous miscarriage at .... weeks - No aborted, miscarriage with medication at .... weeks - No aborted, curettage miscarriage ... weeks - No aborted, abortion at .... weeks |
| --- | --- |
| 2^e^ | Was the pregnancy planned?   - Scheduled - Unscheduled   Did you carry the pregnancy to term (until at least 37 weeks of pregnancy)?   - Yes - No, premature birth at ... weeks. - No aborted, spontaneous miscarriage at .... weeks - No aborted, miscarriage with medication at .... weeks - No aborted, curettage miscarriage ... weeks - No aborted, abortion at .... weeks |
| 3^e^ | Was the pregnancy planned?   - Scheduled - Unscheduled   Did you carry the pregnancy to term (until at least 37 weeks of pregnancy)?   - Yes - No, premature birth at ... weeks. - No aborted, spontaneous miscarriage at .... weeks - No aborted, miscarriage with medication at .... weeks - No aborted, curettage miscarriage ... weeks - No aborted, abortion at .... weeks |
| 4^e^ | Was the pregnancy planned?   - Scheduled - Unscheduled   Did you carry the pregnancy to term (until at least 37 weeks of pregnancy)?   - Yes - No, premature birth at ... weeks. - No aborted, spontaneous miscarriage at .... weeks - No aborted, miscarriage with medication at .... weeks - No aborted, curettage miscarriage ... weeks - No aborted, abortion at .... weeks |
| ... |  |

Did you have a microbial infection during one of your pregnancies?

- Yes
  - If so, which one?
    - Cytomegalovirus (CMV).
    - Toxoplasmosis
    - Other:...........
  - No
  - I don't know

Are you currently breastfeeding?

- Yes
- No

What remedies have you used in the past three months to avoid getting pregnant? Please also indicate which brand you used (multiple answers possible).

- Contraceptive pill:.........................
- Mini pill:.........................
- Lancing pill:.........................
- Contraceptive patch (e.g., Evra) :.........................
- Contraceptive ring (e.g., Nuvaring) :.........................
- Hormonal implant (e.g., Implanon) :.........................
- Hormone IUD (e.g., Mirena): .........................
- Copper IUD:.........................
- Condom:.........................
- Pessary (= rubber dome) :.........................
- Periodic abstinence (= not having sex in fertile period)
- Coitus interruptus (= withdrawing penis before ejaculation)
- Other:.........................
- No

Are you currently using another method?

- No
- Yes, which one?.............................. (same answer options as above)

Why haven't you used a contraceptive method in the last three months?

- I didn't have sex
- I didn't have sex that I could get pregnant from
- I am infertile or reduced fertility
- I would like to become pregnant
- Contraception is expensive
- My partner is infertile or reduced fertility
- My partner uses contraception
- My partner has been sterilized
- I have been sterilized
- I don't like to use
- My partner does not want to use contraception
- The use of contraception is not the norm within my culture
- I do not use contraception for religious reasons
- I have a female partner
- Others:....................

First day of your last period (= first day of monthly bleeding): ........./........./............

Do you currently have your monthly bleeding at regular intervals?

- Yes
- No
- Is this related to any of the following possibilities?
  - I have no/never monthly bleeding
  - I have yet to have monthly bleeding after giving birth
  - I am in my transition period (period just before menopause)
  - I am in my menopause
  - I take my hormonal birth control through
  - I don't know
  - Other: .......

On average, how many days are there between the first day of your period and the first day of your next?

- I do not use hormonal birth control or any other hormonal treatment that may affect my menstrual cycle: ..........
- I use hormonal birth control or another hormonal treatment that may affect my menstrual cycle
- I don't have a menstrual cycle
- I don't know

Do you have pain (e.g., headache, backache, lower abdominal pain, etc.) during monthly bleeding? If yes, also describe what pain you have.

- No, I am never in pain
- Yes, I am always in pain
  - Do you use painkillers to deal with the pain?
- No
- Yes, which and how many per day painkillers ?.........................
- Yes, I have pain sometimes
  - Do you use painkillers to deal with the pain?
- No
- Yes, which and how many a day of painkillers?.........................

Do you experience pain on the days around your ovulation (about14 days after the first day of your period)?

- - Yes
  - No
  - Sometimes
  - Don't know

During my monthly bleeding, I use (multiple answers possible):

- - Tampon
  - Monthly pads
  - Menstrual Cup
  - Panty liners
  - Other: .....................

In the last month, have you experienced one or more symptoms on your vulva or vagina (multiple answers possible)?

- No
- Redness
- Swelling
- Pain (general)
- Itch
- Burning sensation
- Increase in vaginal discharge (other than normal monthly discharge)
- Change in vaginal discharge (different color, unpleasant odor)
- Pain during sexual contact
- Other:...................................
- If an answer designated, indicate how serious
  - No
  - Mild complaints
  - Moderate complaints
  - Serious complaints

Have you ever suffered from one or more of the following conditions? (multiple answers possible)

| Condition | No | I don't know | Yes, now | Yes, sometime |
| --- | --- | --- | --- | --- |
| PCOS (polycystic ovarian syndrome) |  |  |  |  |
| Endometriosis |  |  |  |  |
| Chlamydia |  |  |  |  |
| Gonorrhea |  |  |  |  |
| Bacterial vaginosis |  |  |  |  |
| Aerobic vaginitis |  |  |  |  |
| Herpes |  |  |  |  |
| Mycoplasma |  |  |  |  |
| Mold |  |  |  |  |
| Genital warts |  |  |  |  |
| Trichomonas vaginalis |  |  |  |  |
| Bladder infection |  |  |  |  |
| Other:....... |  |  |  |  |

If yes,

- Were you treated for this? (multiple answers are possible-please indicate for which condition)
  - Yes, through antibiotics, for ................
  - Yes, by antimycotics (product against fungal infections), for ................
  - Yes, through probiotics, for ................
  - Yes, through another product: ................ For ................
  - I don't know
  - No

To your knowledge, have your mother/sister(s)/daughter(s)/other female relatives, already had a similar condition?

- - Yes, what is your relationship?..............................
  - No
  - Don't know

Have you had a change in your lifestyle in the last three months (e.g., moving, new partner, change in diet, change in exercise, stressful periods, etc.)?

- - Yes, specifically ....................................
  - No
  - Don't know

Which of the following products have you ever used? (multiple answers possible)

- Vaginal antimycotic (e.g., Gynodaktarin, Clotrimazole, Canestene, etc.)
  - How often? Several times a day / daily / weekly / monthly / rarely / no more

- Are you still using this product?

- - - Yes
    - No
- Vaginal soap (e.g. Lactacyd femina, Multi-Gyn, Sebamed, ...)
  - How often? Several times a day / daily / weekly / monthly / rarely / no more

- Are you still using this product?

- - - Yes
    - No
- Vaginal probiotics (e.g. Physioflor, Pro-Ven, Mucogyne, Gynoflor, ... )
  - How often? Several times a day / daily / weekly / monthly / rarely / no more

- Are you still using this product?

- - - Yes
    - No
- None of the above

Have you ever had a vaginal swab taken by the doctor or gynecologist?

- Yes
- No
- Don't know

Have you been vaccinated against HPV (human papillomavirus)?

- Yes
- No
- Don't know

In some cultural/religious communities, girls and women are circumcised. Does this happen in your community?

- Yes
- No
- Don't know

Have you undergone female circumcision?

- Yes
- No

Have you ever had surgery on your vagina or vulva? We do not mean the uterus or ovaries here.

- Yes
- No

Have any operations been performed on your female genitalia for non-medical reasons (e.g., punctures, piercing, tattooing, etc.)?

- Yes
- No

Have you ever worried about your vaginal health?

- Yes
- No
- A little

With whom do you feel you can talk openly about your vaginal health? (multiple answers possible)

- Partner
- Friends/friends
- Children
- Colleagues
- GP
- Gynecologist
- Other: ...
- Nobody

**Personal hygiene**

Thinking about the past three months, can you indicate how often you did the actions below?

| Question | Daily | More than 3x/week | Weekly | Monthly | Rarely | Never |
| --- | --- | --- | --- | --- | --- | --- |
| How often do you take a shower? |  |  |  |  |  |  |
| How often do you use body lotion/milk? |  |  |  |  |  |  |
| How often do you use hand cream? |  |  |  |  |  |  |
| How often do you use face cream? |  |  |  |  |  |  |
| How often do you use exfoliators or scrubs for your skin (face/body)? |  |  |  |  |  |  |
| How often do you use antibacterial soap or disinfectants for your hands? |  |  |  |  |  |  |
| How often do you use medicinal creams (e.g., cortisone or antibiotic creams) |  |  |  |  |  |  |
| How often do you use sunscreen? |  |  |  |  |  |  |
| How often do you use perfume? |  |  |  |  |  |  |
| How often do you use deodorant? |  |  |  |  |  |  |
| How often do you use shaving soap/shaving products? |  |  |  |  |  |  |

Do you have sensitive skin?

- Yes
- No

What skin products do you normally use **daily**? (multiple answers possible)

- Cosmetics
- Non-medicated creams (e.g., day cream, body lotion, etc.)
- Medicinal creams
- Soaps
- Deodorants
- Perfume
- Scrub
- Other:.....................
- No

Do you suffer from excessive sweating?

- Yes
- No

Does this coincide with specific period in your menstrual cycle?

- Yes
- No
- Don't know

How often do you shave your pubic area?

- Daily
- More than 3x/week
- Weekly
- Monthly
- Rarely
- Never

During the last three months, have you vaginally flushed (by injecting a liquid into your vagina - also known as vaginal showering)?

- Yes
- No

Studies have shown that certain ties (kinship, cohabitation, ...) between individuals can affect the microbiome. Therefore, we would also like to investigate this with Isala. Do you agree that your results may be linked to someone in your family or with whom you live who is also participating in Isala? Provided both of you have given permission. You will not be able to see each other's answers or results.

- I agree
- I do not agree

Does anyone in your extended family circle or that you live with participate in Isala? If yes, please also indicate the number of people involved.

- Yes: how much? ...
- No

(If yes) List the name and relationship relationship with this person in the table below.

|  |  | In this way, we are related: |
| --- | --- | --- |
| First name | Name |  |
|  |  |  |
|  |  |  |

We asked a lot about your lifestyle factors and your habits over the past 3 months but we know that the coronavirus had a big effect on many lives and habits. Anything you want to say about it?

..............................................................................................................................

..............................................................................................................................

..............................................................................................................................

Do you have any comments for us after completing this questionnaire?

..............................................................................................................................

..............................................................................................................................

..............................................................................................................................

**Confidentiality**

The protection of personal data is determined by law by the Privacy Commission. All information is kept confidential in accordance with the law on privacy. Participants will be identified by code. If the results of this study are published in a report or scientific journal, no name will be mentioned.

**Short follow-up QUESTIONNAIRE**

Version 1 - July 13, 2020

**Integrated genetic and functional analysis of the female microbiome in a Flemish cohort**

Completing this additional questionnaire will take about 5 minutes. After all, because of the Corona crisis and its impact on our lifestyle, it appeared necessary to ask some additional questions or to take a quick poll to see if things might have changed. The survey is still designed so that your personal data cannot be directly linked to your answers to ensure your privacy. Be sure to give us a call if you have additional questions while completing the questionnaire by contacting us at isala@uantwerpen.be.

**General**

Weight: ............kg

Has your sexual relationship status changed recently?

- No, I have the same sexual partner
- Yes, I have another sexual partner
- Yes, I have multiple sexual partners
- Yes, I no longer have a sexual partner

Can you make ends meet monthly with the sum of total disposable family income (all included^[[3]](#footnote-4)^ )?

- Very difficult
- Difficult
- Rather difficult
- Rather easy
- Easy
- Very easy

In 2020, what was the total disposable household income (all inclusive^2^ ) per month (in a normal month - outside the COVID-19 period such as January or February 2020)?

- Amount approximately per month in euros:............
- I don't know
- I don't want to say

How many people depend on this income?.............

How large is your share of the family income?

- I am the only one contributing
- I have the largest share
- We contribute equally
- I contribute the least
- I do not contribute

**Diet**

Did you take probiotics or yogurt with live ferments (e.g. Pur Natur) 24h before taking your vaginal swab?

- Yes, which one? .................
- No

Did you drink so much in the last **24 hours** that you had to get up at night to pee?

- Yes
- No

How many servings did you eat or drink of the items listed below in the past **24h** (serving = a handful or glass)?

|  | No | 1 serving | 2 servings | 3 servings | More than 3 servings |
| --- | --- | --- | --- | --- | --- |
| Dairy products (cheese, milk, yogurt, buttermilk, etc.) |  |  |  |  |  |
| Fermented foods based on lactic acid bacteria (olives, salami, sauerkraut, kimchi, pickles, ... ) |  |  |  |  |  |
| Alcohol |  |  |  |  |  |
| Meat |  |  |  |  |  |
| Fish |  |  |  |  |  |
| Coffee |  |  |  |  |  |
| Sweet drinks with sugar (e.g., soda, fruit juice) |  |  |  |  |  |
| Sugar-free sweet drinks (e.g., light sodas) |  |  |  |  |  |
| Fruits high in fiber or possible prebiotics: bananas, kiwi, nectarine, peach, persimmon, raspberries, apples, pears, oranges, pomegranate, grapefruit, cranberries (also dried), prunes, raisins |  |  |  |  |  |
| Vegetables high in fiber and possible prebiotics: onion, chicory, asparagus, garlic, leeks, peas, beans, artichokes, savoy cabbage, avocado |  |  |  |  |  |
| Cold pasta (source of resistant starch) |  |  |  |  |  |
| Whole wheat bread |  |  |  |  |  |
| sourdough bread |  |  |  |  |  |
| Quinoa |  |  |  |  |  |
| Chia seeds, flaxseeds, pumpkin seeds, poppy seeds, sunflower seeds |  |  |  |  |  |
| Nuts (almonds, cashew, pistachio, walnuts, hazelnuts ...) - no salted bran nuts |  |  |  |  |  |
| Chocolate |  |  |  |  |  |
| Candy and other sweets |  |  |  |  |  |
| Chips, salty cookies and crackers |  |  |  |  |  |

Do you believe that the use of probiotics (= live bacteria that by definition are beneficial to your health when taken in sufficient amounts) can be beneficial to your health?

- Yes
- No
- I don't know

Have you ever taken probiotics (orally, vaginally or in some other form)?

- Yes
- No

If yes, how did you come into contact with probiotics?

- Recommended by a physician
- Recommended in pharmacy
- Online/media (TV, radio) information
- Friends, family, other
- Other: .........

What did you use probiotics for? (multiple answers possible)

- Immune System
- Digest
- Vaginal health
- Stress/mental health
- Allergy
- Skin
- Respiratory diseases
- Other: ..............

What probiotic products did you use? Please also provide the name of the product
................................

**General health**

Has your general health status changed?

- Yes, please specify:................
- No

Have you had any antibiotic or antifungal treatment in the **past month** (also report vaginal antibiotics and antifungal such as e.g. gynodaktarin)?

- Yes, which one?..................
- No

Have you taken any new medication in the **last month**?

- Yes, which one?
- No

Did you feel gloomier and/or more stressed than usual on average over the **past month**?

- Yes
- Same
- Less than previously indicated

**Reproductive and sexual health**

At what age did your monthly bleeding start?

- Age in years: ............
- I don't know

Did you have sex in the **past week**? By this we mean all kinds of stimulation of the vagina, vulva, clitoris and anus, including manual, oral, vaginal and anal sex, masturbation,...(with or without vaginal penetration)

- Yes
- No

If yes, in the past **24h** have you received or performed the following sexual acts on yourself (masturbation)?

- Manual sex
- Oral sex
- Anal sex
- Vaginal sex
  - Without penetration
  - Penetration with penis
  - Penetration with finger(s)/hand
  - Penetration with toys
  - Penetration with other: ...
- Other: ....

Are you currently breastfeeding?

- Yes
- No

What drug have you used in the **past month** to avoid getting pregnant? Please also indicate which brand you used (multiple answers possible)

- Contraceptive pill:.........................
- Mini pill:.........................
- Lancing pill:.........................
- Contraceptive patch (e.g., Evra) :.........................
- Contraceptive ring (e.g., Nuvaring) :.........................
- Hormonal implant (e.g., Implanon) :.........................
- Hormone IUD (e.g., Mirena): .........................
- Copper IUD:.........................
- Condom:.........................
- Pessary (= rubber dome) :.........................
- Periodic abstinence (= not having sex in fertile period)
- Coitus interruptus (= withdrawing penis before ejaculation)
- Other:.........................
- No

Why haven't you used a contraceptive method in the past month? (multiple answers possible)

- I didn't have sex
- I had sex but couldn't get pregnant from it
- I am infertile or reduced fertility
- I would like to become pregnant
- Contraception is expensive
- My partner is infertile or reduced fertility
- My partner uses contraception
- My partner has been sterilized
- I have been sterilized
- I don't like to use
- My partner does not want to use contraception
- The use of contraception is not the norm within my culture
- I do not use contraception for religious reasons
- I have a female partner
- Other:....................

Have you had a period in the last 6 months?

- Yes
- No
- I don't know

If yes, when was your first day of your last period?

- The first day of my last period was (dd/mm/yyyy): ........./........./.........
- I don't know

Have you used the following products in the **past 48h**?

- - Tampon
  - Monthly pads
  - Menstrual Cup
  - Panty liners
  - Other: .....................
  - No

Have you experienced one or more complaints of your vagina or genitourinary system **in the last week** (multiple answers possible)?

- No
- Redness
- Swelling
- Pain (general)
- Itch
- Burning sensation
- Increase in vaginal discharge (other than normal monthly discharge)
- Change in vaginal discharge (different color, unpleasant odor)
- Pain during sexual contact
- cystitis
- Other:...................................

Which of the following products have you used in the **past 48h**? (multiple answers possible)

- Vaginal soap (e.g. Lactacyd femina, Multi-Gyn, Sebamed, ...)
- Vaginal probiotics (e.g. Physioflor, Pro-Ven, Mucogyne, Gynoflor, ...)
- Vaginal Shower
- None of the above

**Personal hygiene**

| **Have you spent the last 24 hours?** | **Yes** | **No** |
| --- | --- | --- |
| Took a bath? |  |  |
| Taken a shower? |  |  |
| Only washed your vaginal area at the sink, bidet, Japanese toilet, etc.? |  |  |
| Wet wipes used? |  |  |
| Slept at night with your underwear on? |  |  |

How do you clean your vagina after going to the toilet ?

- Wiping from front to back
- Wiping from back to front
- I do both
- Without wiping
- Other:............

After a toilet visit, I use:

- Mostly toilet paper
- Usually a damp cloth
- Mostly water and my hands
- Mostly a washcloth
- Other: ............

What underpants have you worn in the **past week** (Multiple answers possible)?

- Satin
- Cotton
- Synthetic, Lycra
- Synthetic, Polyamide
- Synthetic, Polyester
- Synthetic, I don't know
- Bamboo
- Other:............
- Don't know

How regularly do you change your underpants?

- Several times a day
- Every day
- Several times a week
- Every week
- Less than once a week
- I don't wear underwear

Did you go swimming in the **24 h** before taking your vaginal swab?(Multiple answers possible0

- Yes, Private pool
- Yes, Public Pool
- Yes, Sea
- Yes, Swimming Pond
- Yes, River
- Yes, Jacuzzi
- Yes, Other: .........
- No

From your own experience, do you have anything you want to share that you think is important for your vaginal health? Your tips & tricks for getting rid of a recurring vaginal infection? Topics you think are important that we haven't yet addressed/questioned?

..............................................................................................................................

..............................................................................................................................

Do you have any other comments for us after completing this questionnaire?

..............................................................................................................................

..............................................................................................................................

**Confidentiality**

The protection of personal data is determined by law by the Privacy Commission. All information is kept confidential in accordance with the law on privacy. Participants will be identified by code. If the results of this study are published in a report or scientific journal, no name will be mentioned.

1. Your personal study number can be found in mail or on your personal account on the isala website [↑](#footnote-ref-2)
2. Antimycotics = drugs with an antifungal (fungistatic) or fungicidal (fungicide) effect e.g. Daktarin, Lamisil, Canesten,... [↑](#footnote-ref-3)
3. The living conditions of a household obviously depend to a large extent on income. This is the total disposable income of the various members of the household combined. Multiple members can therefore contribute. The total disposable income of a household consists of, (1) net wages, salaries and net business incomes for the self-employed, (2) social benefits and child benefits, (3) additional incomes such as rental income, interest and the like. The sum of all these incomes for all persons in your household is the total disposable income of your household. [↑](#footnote-ref-4)
