# Supplementary material for: Salivary microbiome of healthy women of reproductive age
Source: mBio. 2023 Sep 1;14(5):e00300-23. doi: 10.1128/mbio.00300-23 (PMC10653790; doi:10.1128/mbio.00300-23)
Supplement: Table S1 — Demographics of non-responders. This information is based on all participants that did not fill in the questionnaire accompanying the sample and/or did not send in a saliva sample. [file mbio.00300-23-s0003.docx]

|  | **Total (n = 65)** | |
| --- | --- | --- |
|  | **[n]** | **[%]** |
| **Age (years)** |  |  |
| - **Range** | 21 - 50 | / |
| - **Mean** ± **SD** | 32.3 ± 8.4 | / |
| **Body Mass Index (BMI, kg/m²) [mean** ± **SD]** | 25.6 ± 7.7 | / |
| **Obesity (BMI ≥ 30)** | 14 | 21.54 |
| **Underweight (BMI < 18.5)** | 6 | 9.23 |
| **Born in Belgium** | 56 | 86.15 |
| **Current smoker** | 8 | 12.3 |
| **HPV vaccinated** | 22 | 33.85 |
